# Deleterious and protective effects of epothilone-D alone and in the context of amyloid β- and tau-induced alterations

**DOI:** 10.3389/fnmol.2023.1198299

**Published:** 2023-10-12

**Authors:** Ángel Abdiel Robles-Gómez, Benito Ordaz, Jonathan-Julio Lorea-Hernández, Fernando Peña-Ortega

**Affiliations:** ^1^Instituto de Neurobiología, UNAM Campus Juriquilla, Querétaro, Mexico; ^2^Posgrado en Ciencias Biológicas, UNAM, Ciudad Universitaria, México City, Mexico

**Keywords:** Alzheimer’s disease, tau and phospho-tau protein, amyloid - **β**, microtubule, therapeutic, epothilone-D, CA1 pyramidal neurons, temporoammonic pathway

## Abstract

Amyloid-β (Aβ) and hyperphosphorylated tau (P-tau) are Alzheimer’s disease (AD) biomarkers that interact in a complex manner to induce most of the cognitive and brain alterations observed in this disease. Since the neuronal cytoskeleton is a common downstream pathological target of tau and Aβ, which mostly lead to augmented microtubule instability, the administration of microtubule stabilizing agents (MSAs) can protect against their pathological actions. However, the effectiveness of MSAs is still uncertain due to their state-dependent negative effects; thus, evaluating their specific actions in different pathological or physiological conditions is required. We evaluated whether epothilone-D (Epo-D), a clinically used MSA, rescues from the functional and behavioral alterations produced by intracerebroventricular injection of Aβ, the presence of P-tau, or their combination in rTg4510 mice. We also explored the side effects of Epo-D. To do so, we evaluated hippocampal-dependent spatial memory with the Hebb–Williams maze, hippocampal CA1 integrity and the intrinsic and synaptic properties of CA1 pyramidal neurons with the patch-clamp technique. Aβ and P-tau mildly impaired memory retrieval, but produced contrasting effects on intrinsic excitability. When Aβ and P-tau were combined, the alterations in excitability and spatial reversal learning (i.e., cognitive flexibility) were exacerbated. Interestingly, Epo-D prevented most of the impairments induced Aβ and P-tau alone and combined. However, Epo-D also exhibited some side effects depending on the prevailing pathological or physiological condition, which should be considered in future preclinical and translational studies. Although we did not perform extensive histopathological evaluations or measured microtubule stability, our findings show that MSAs can rescue the consequences of AD-like conditions but otherwise be harmful if administered at a prodromal stage of the disease.

## Introduction

Tauopathies are neurodegenerative diseases that involve the hyperphosphorylation, deposition, and seeding of the microtubule-associated protein tau (Mondragón-Rodríguez et al., 2020). Alzheimer’s disease (AD) is the most prevalent tauopathy that also involves the pathological actions of other peptides (Peña-Ortega, 2019; Mondragón-Rodríguez et al., 2020). For instance, aggregated amyloid-β peptide (Aβ) species and hyperphosphorylated tau (P-tau) are the most prominent AD biomarkers (Peña-Ortega, 2019; Mondragón-Rodríguez et al., 2020). Aβ and P-tau complex interactions produce synergistic but also antagonistic effects that can better explain the complex pathophysiology of AD than their independent toxic properties (Ittner et al., 2010; Zempel et al., 2010; Zempel and Mandelkow, 2012). When evaluated in animal models both Aβ (Busche et al., 2012, 2019; Lo et al., 2013; Peña-Ortega, 2013, 2019; Yetman et al., 2016; Angulo et al., 2017; Pickett et al., 2019; Ranasinghe et al., 2022; Barendrecht et al., 2023; Capilla-Lopez et al., 2023) and P-tau (Lo et al., 2013; Yetman et al., 2016; Booth et al., 2016a,b; Angulo et al., 2017; Hatch et al., 2017; Busche et al., 2019; Pickett et al., 2019; Ranasinghe et al., 2022; Barendrecht et al., 2023; Capilla-Lopez et al., 2023) produce diverse or even contrasting effects on neural networks (Lo et al., 2013; Yetman et al., 2016; Angulo et al., 2017; Busche et al., 2019; Pickett et al., 2019; Ranasinghe et al., 2022; Barendrecht et al., 2023; Capilla-Lopez et al., 2023). Thus, the specific effects of tau and Aβ, their possible synergistic (Zempel and Mandelkow, 2012; Busche et al., 2019) and antagonistic actions (Yetman et al., 2016; Ranasinghe et al., 2022; Barendrecht et al., 2023; Capilla-Lopez et al., 2023), as well as their net consequences on brain function are far from being understood and should be evaluated under similar experimental conditions. We are aware that similar attempts have rendered complex results in different experimental (Lo et al., 2013; Yetman et al., 2016; Angulo et al., 2017; Busche et al., 2019; Pickett et al., 2019; Barendrecht et al., 2023; Capilla-Lopez et al., 2023) and clinical settings (Ranasinghe et al., 2022), but we are convinced that this type of experimental approach is required to fully understand the complexity of AD pathophysiology.

Neuronal microtubules are cytoskeletal protein complexes of α- and β-tubulin dimers that influence neuronal morphology, polarity, axonal transport, intrinsic and synaptic properties, as well as several brain functions including learning and memory (Uchida et al., 2014; Hatch et al., 2017; Babu et al., 2020; Guo et al., 2020; Peña-Ortega et al., 2022). Microtubule alterations have been closely associated with AD, mainly due to tau dysfunction, which drives aberrant microtubule dynamics *in vivo* and *in vitro* (Qu et al., 2017; Peña-Ortega et al., 2022). Recently, Peris et al. (2022) demonstrated that premature microtubule longevity is an early stage of cellular AD that impairs synapse function. The presence of Aβ_42_ oligomers also disorganizes microtubule bundles in neurons through a tau-dependent mechanism (Zempel et al., 2010; Golovyashkina et al., 2015). However, Aβ exposure causes microtubule stabilization in cultured hippocampal neurons (Qu et al., 2017). Despite these apparent discrepancies, the modulation of microtubule dynamics towards more stable states has been proposed as a potential treatment for AD (Brunden et al., 2010; Guo et al., 2020; Peña-Ortega et al., 2022). Microtubule stabilizing agents (MSAs) can be beneficial, but their use must be carefully considered because they can also induce tau hyperphosphorylation and spine loss in cultured hippocampal neurons (Qu et al., 2017), and have other side effects (Wefel and Schagen, 2012; Atarod et al., 2015; Golovyashkina et al., 2015; Clark et al., 2020). MSAs are commonly used in chemotherapy as cytotoxic drugs (Cavaletti and Marmiroli, 2010) and, in recent years, their administration to AD animal models has yielded positive results (Brunden et al., 2010; Guo et al., 2020). However, tilting microtubule dynamics, by the administration of either MSAs or MDAs (microtubule destabilizing agents), has clear deleterious effects on neuronal survival (Chiorazzi et al., 2009) and morphology (Golovyashkina et al., 2015; Qu et al., 2017; Clark et al., 2020), axonal transport (Clark et al., 2020), brain anatomy and cognition (Wefel and Schagen, 2012; Atarod et al., 2015), in otherwise healthy individuals. Thus, the neurophysiological consequences of MSAs depend on the state of the system (i.e., normal or pathological conditions) and require proper testing in each physiological and pathological condition. In this study we used behavioral and electrophysiological approaches to characterize the pathological consequences of Aβ, P-tau, and their combination in rTg4510 mice, on hippocampal function. Furthermore, we characterized the effects of epothilone-D (Epo-D), an MSA that penetrates the blood–brain barrier (Brunden et al., 2010), in these pathological conditions as well as its effects in heathy individuals. The cognitive evaluation was done with the Hebb-Williams (HW) test, which has been extensively used in different experimental settings (Rogers and Kesner, 2003; Lee and Kesner, 2004; Jerman et al., 2006; Vago et al., 2007; Hunsaker et al., 2008; Churchwell et al., 2010; Vidal-Infer et al., 2012; Boutet et al., 2018; Méndez-Salcido et al., 2022) and seems to be more sensitive to neural alterations than other common water or radial mazes (Pereira et al., 2005). Furthermore, the performance during the different phases of this test has been closely related to specific microcircuits (Rogers and Kesner, 2003; Lee and Kesner, 2004; Jerman et al., 2006; Vago et al., 2007; Hunsaker et al., 2008; Churchwell et al., 2010). It can be performed under low-stress conditions (Pritchett and Mulder, 2004) and can measure cognitive flexibility by changing the maze configuration (Shore et al., 2001; Vidal-Infer et al., 2012; Boutet et al., 2018). Moreover, the preclinical results derived from this maze can be directly compared to human health and disease (Shore et al., 2001; MacLeod et al., 2010; Boutet et al., 2018). We demonstrated that Epo-D can rescue various cognitive and functional alterations induced by Aβ, P-tau, and their combination, although we did not perform extensive histopathological evaluations. However, Epo-D also has detrimental effects, even in control conditions, which preclinical and clinical studies should consider when using MSAs to treat AD or similar pathologies.

## Materials and methods

### Animals

**Figure 1:**
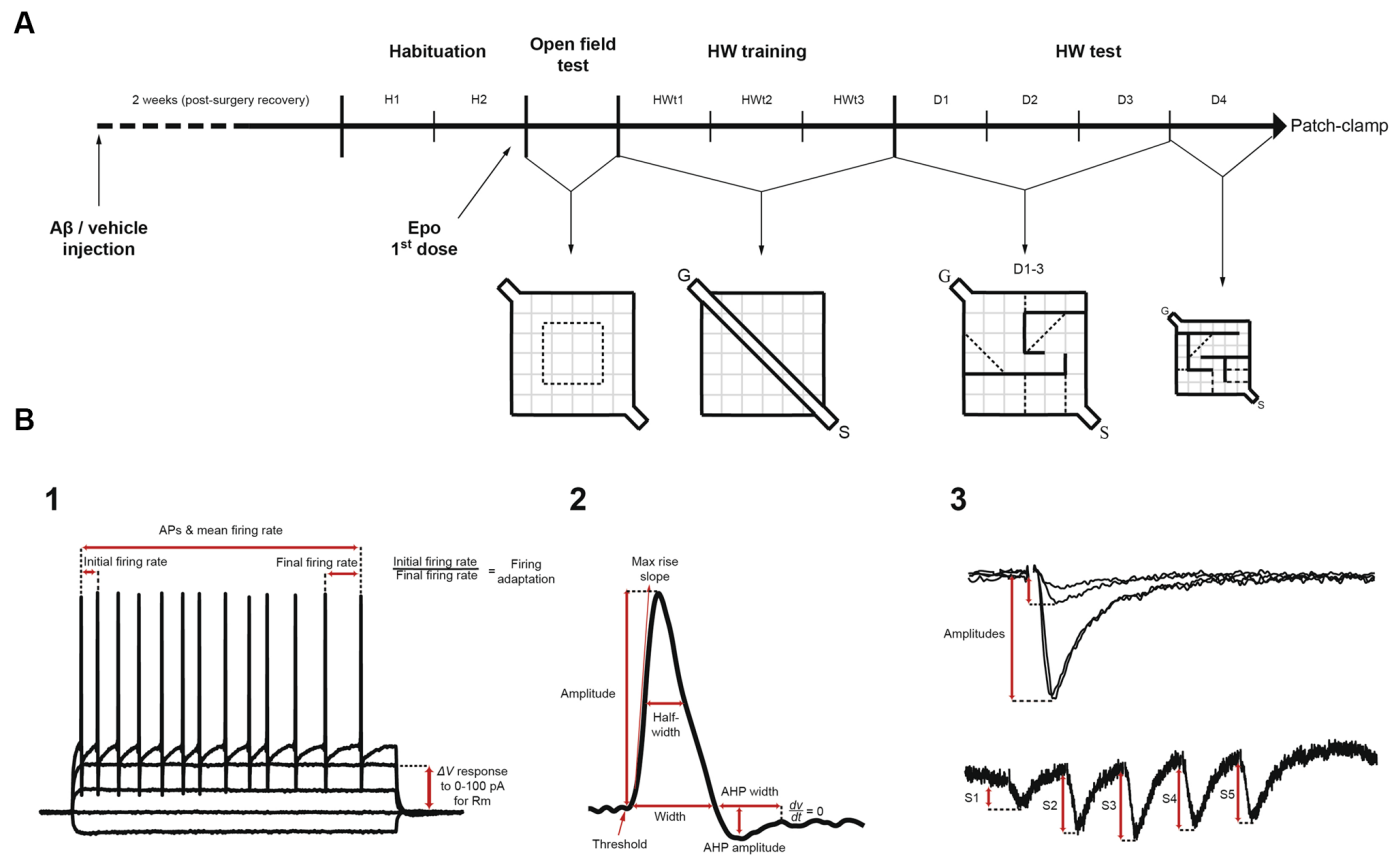
Experimental design and electrophysiological measurements. **(A)** Mice were given an intracerebroventricular injection of either amyloid-β (Aβ) or vehicle (F12 medium). Two weeks after surgery (recovery), mice were habituated for 2 days (H1 and H2) to the context and the experimenter, before receiving the first dose of Epothilone-D (Epo-D) or its vehicle (DMSO). Subsequently, mice were tested in the open field test once. Next, mice were trained in a linear corridor located in the Hebb-Williams (HW) arena for three consecutive days (HWt1-3). Then, animals were challenged in the HW test with the standard #4 maze for three consecutive days (D1-3). On day 4 (D4), the maze was replaced with the standard #9 maze. After the behavioral tests, these mice were used for the electrophysiological recordings (patch-clamp). **(B)** Pyramidal neurons were recorded with standard patch-clamp protocols. **(1)** To analyze passive and active membrane properties we used I/V and I/F curves. **(2)** Action potential characteristics were measured. **(3)** Excitatory postsynaptic currents induced by temporoammonic pathway stimulation were measured through I/O curves and short-term plasticity train protocols. S = start compartment, G = goal compartment, AP = action potential, Rm = membrane input resistance, AHP = afterhyperpolarization, S1-5 = excitatory postsynaptic currents 1 to 5.

The experimental procedures were reviewed and approved by the Local Research Ethics Committee (INB-UNAM). We used C57BL/6 wild-type (WT) and rTg4510 transgenic (Tg) mice (SantaCruz et al., 2005; The Jackson Laboratory Strain 024854). Tg mice overexpress the human microtubule-associated protein tau (MAPT) gene bearing the mutation P301L, whose expression is controlled by the calcium/calmodulin-dependent protein kinase II (CaMKII) promoter (SantaCruz et al., 2005). Genotyping was performed by a standard PCR assay (The Jackson Laboratory Strain 024854). Tg and WT mice (5–6 months old; Table 1) were housed in same-sex pairs. Animals were maintained in a 12 h light–dark cycle with food and water available *ad libitum*. At 5–6 months old Tg mice already exhibit clear cognitive impairment and tau pathology (Ramsden et al., 2005; SantaCruz et al., 2005; Yue et al., 2011). This age is also characterized by the onset of neurodegeneration and mild motor impairments (Ramsden et al., 2005; SantaCruz et al., 2005; Camargo et al., 2021). Male and female mice were used in all procedures (Table 1). There are contradictory reports regarding sexual dimorphisms in the Tg mice: whereas there is a report of Tg females exhibiting more tau pathology and cognitive impairment than males (Yue et al., 2011), there is a contrasting report of Tg males exhibiting more tau pathology as well as increased olfactory and motor deficits than females (Camargo et al., 2021). In our sample, only two variables showed differences between males and females: the center index (quantified in the open field test) and the after hyperpolarization amplitude (data not shown). Animals were subjected to an experimental protocol described in Figure 1A that sequentially included a microinjection of Aβ/vehicle, administration of Epo-D/vehicle, the open field test, the Hebb–Williams (HW) test, and patch-clamp recordings (Figure 1). All experimental conditions will be described next.

**Table 1:** Summary of the behavioral (as # of animals) and electrophysiological (as # of neurons) measurement divided by sex.

	Behavior			Electrophysiology		
	M	F	Total	M	F	Total
WT veh	4	3	7	5	5	10
WT Epo	4	4	8	4	4	8
WT Aβ	3	4	8/7	5	4	9
WT Aβ + Epo	3	4	7	5	6	11
Tg veh	4	5	10/9	3	5	8
Tg Epo	4	5	9	3	5	8
Tg Aβ	3	4	7	4	5	9
Tg Aβ + Epo	5	3	8	5	4	9
Total	30	32	64/62	34	38	72

Note that for both groups, a couple of mice are not included in all behavioral tests because they did not reach the inclusion criteria for the Hebb-Williams test.

### Surgery and microinjection

For intracerebroventricular (ICV) injection of Aβ or its vehicle, mice were induced into anesthesia with ketamine and xylazine (90 and 10 mg/kg, respectively) and maintained with sevoflurane 0.4% during surgery. Anesthesia was verified by the absence of tail pinch responses. After the animals were anesthetized, they were gently mounted on a stereotaxic apparatus. Once the skull was exposed, a hole was drilled with the following coordinates (from Bregma): −1 mm medio-lateral, −0.25 antero-posterior. A microinjector was gently introduced into the lateral ventricle (2.8 mm from the skull) and a total of 5 μL of either the Aβ oligomers solution (500 pmoles; Martínez-García et al., 2021) or its vehicle (F12 medium; Balleza-Tapia et al., 2010; Hernández-Soto et al., 2019) was injected at 0.5 μL/min with a Hamilton syringe. Martínez-García et al. (2021) and Desbène et al. (2012) have shown that this ICV dose of Aβ is enough to induce cognitive and neurophysiological alterations in mice (Desbène et al., 2012; Martínez-García et al., 2021). Intracerebral Aβ leads to its long-lasting non-fibrillar accumulation in the tissue (Alvarado-Martínez et al., 2013; Zussy et al., 2011, 2013; Sharma et al., 2016; Torres-Flores and Peña-Ortega, 2022). After the microinjection, mice were sutured and given meloxicam (2 mg/kg) to reduce inflammation and pain. All animals were returned to their home cages and allowed to recover from surgery for 2 weeks before any further manipulation. Mice that received an ICV injection of Aβ are also referred to as subjects with “global Aβ” (Torres-Flores and Peña-Ortega, 2022).

### Epo-D administration

Epo-D (MedChem Express) was dissolved in DMSO and aliquoted in stocks of 1 mg/mL (2.057 mM), which were stored at −70°C. This solution was intraperitoneally injected to mice (2 mg/kg; Brunden et al., 2010). Upon this administration Epo-D reach a nanomolar concentration in the brain (Schering AG patent, WO 03/074053 A1, published 12/09/2003; Brunden et al., 2010) that lasts for several days after the administration (Brunden et al., 2010). The first dose was injected 24 h before the open field test (Figure 1A), and then applied weekly (Figure 1A).

### Open field test

**Figure 2:**
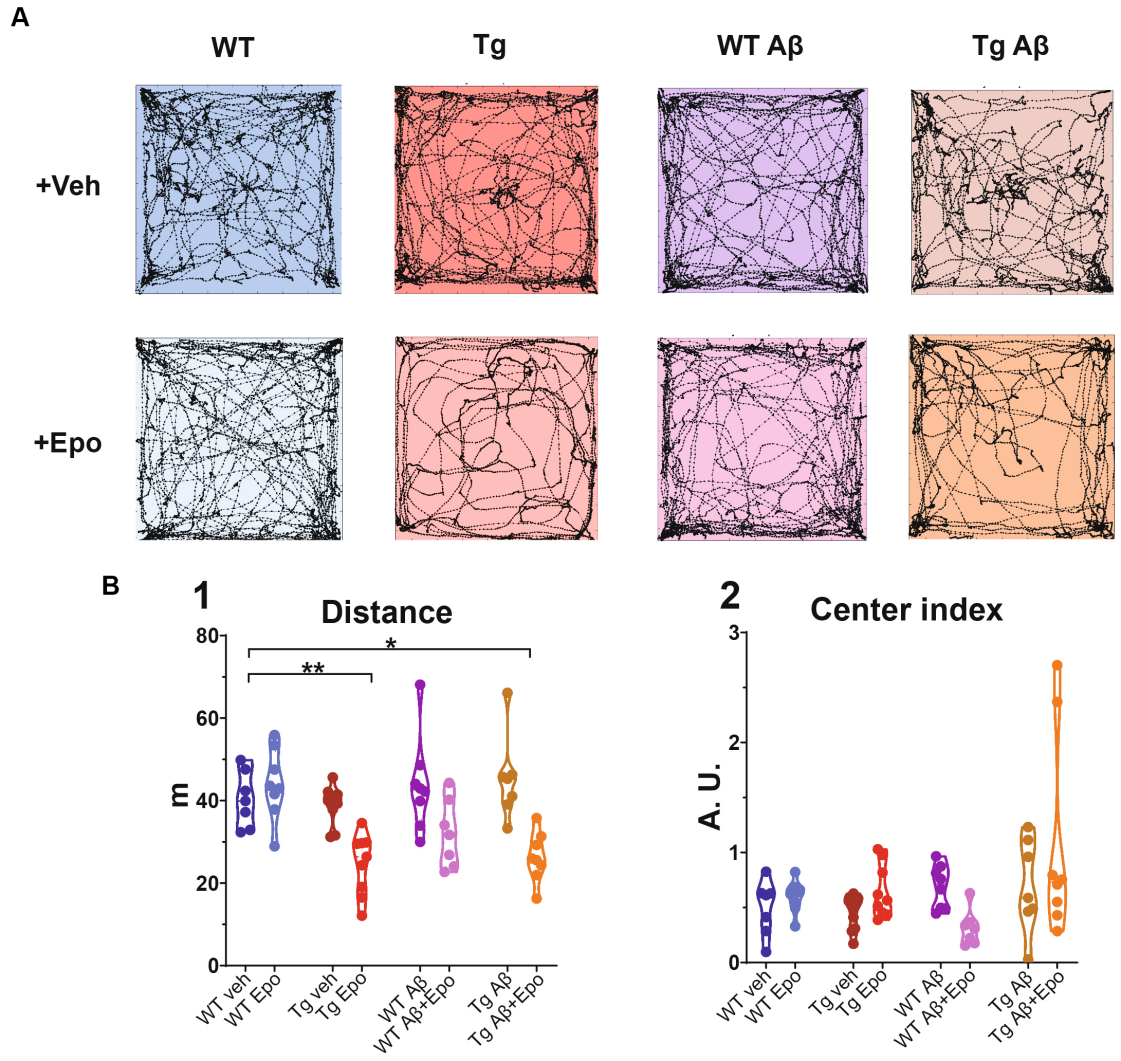
Open field test. **(A)** Trajectories of representative mice from each experimental group. The dimensions of the open field arena are 60 × 60 cm. **(B)** Quantification of the **(1)** total traveled distance (in meters; m) and **(2)** center index (see Material and Methods). WT = wild-type mice, Epo = Epothilone-D, Aβ = amyloid-β, Tg = transgenic mice, veh = vehicle, A.U. = arbitrary units. Asterisks denote significant differences as follows: *p < 0.05, **p < 0.01.

Spontaneous exploration was assessed with the open field test (Salgado-Puga and Pena-Ortega, 2015). Animals were handled and habituated to the context and the experimenter in 10 min sessions for 2 days, two sessions per day, prior to testing (H1 and H2; Figure 1A). On the third day (Figure 1A), animals were introduced into a novel 60 × 60 × 30 cm square field and their spontaneous behavior was video-recorded for 5 min (Logitech Webcam). Of the 3,600 cm^2^ comprising the arena, a square of 900 cm^2^ (30 × 30 cm) was considered its center (25% of the total area; Figures 1A, 2). Thus, we calculated the proportion of time spent in the center as follows:


$$CI=\frac{25\%}{\%\mathrm{time}}$$

Where *CI* is the center index and *% time* is the proportional time spent in the central area with respect to the total exploration time (5 min). Values below 1 indicate that mice preferred to explore the periphery.

### Hebb–Williams test

**Figure 3:**
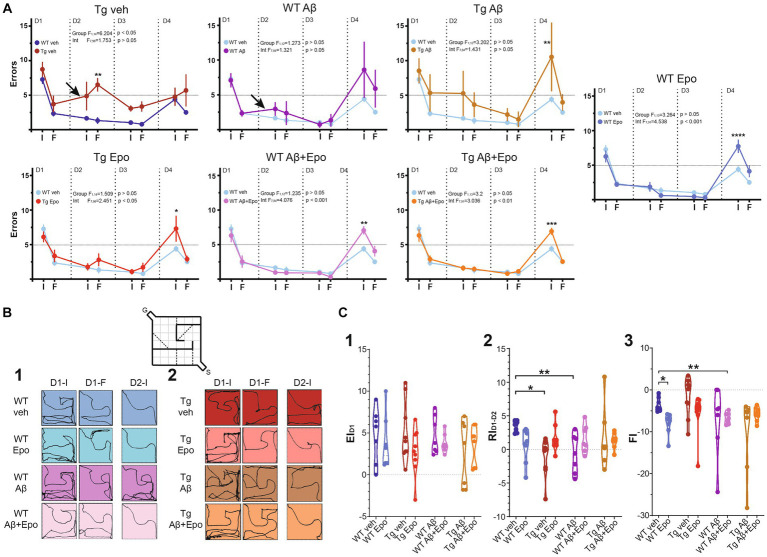
Hebb-Williams test. **(A)** Performance (as # of errors) of the WT mice and Tg mice during the 4 days testing. The performance was averaged for the errors in the initial (I) and final (F) five trials of each day. The control group (WT veh; dark blue) was included in the graph with the Tg animals for a direct comparison. It was also included in the other graphs (light blue) for the same reason. The arrows indicate the two cases in which retrieval was affected (note the positive slope produced by an increase in errors compared to the previous day). **(B)** Trajectories of representative trials of each group at the beginning and end of day one [**(1)**; D1-I and D1-F, respectively; i.e., encoding phase of the test] and at the beginning of day two [**(2)**; D2; i.e. retrieval phase of the test]. The inset represents the configuration of the #4 HW maze. **(C)** Quantification of the encoding [**(1)**; EI_D1_], retrieval [**(2)**; RI_D1-D2_] and flexibility [**(3)**; FI, 3] indexes (see Material and Methods). WT = wild-type mice, Epo = Epothilone-D, Aβ = amyloid-β, Tg = transgenic mice, veh = vehicle. Asterisks denote significant differences as follows: *p < 0.05, **p < 0.01, ***p < 0.001, ****p < 0.0001.

The day after the open field test, animals were trained in a modified version of the HW test (Méndez-Salcido et al., 2022; Figures 1A, 3). First, mice were trained in a linear corridor for three consecutive days (HWt1-3; Figure 1A), with 10 assays per day (i.e., 1 session). In each assay the animal was left in the starting compartment (S) to walk through the delimited corridor, reach the goal compartment (G), and get a reward (a small piece of corn flake; Figure 1A). After each session, mice were allowed to eat freely for 6 h and then maintained under an 18 h food deprivation regime before the next training day (18–6 h cycle). Animals that did not reach the goal compartment within 5 s in the last five assays of the third training day were discarded (only two out of 64 animals; Table 1).

The standard #4 HW maze (MacLeod et al., 2010; Figures 1A, 3B), previously tested in our laboratory (Méndez-Salcido et al., 2022), was used for three consecutive days (D1-3). The test consisted in one encoding phase (on D1) and two retrieval phases (on D2 and D3). Then, on D4, a different maze was used (#9 HW maze; MacLeod et al., 2010) to evaluate the subject’s cognitive flexibility or reversal learning (Torres-Flores and Peña-Ortega, 2022). Considering that both mazes include five imaginary error lines (see Figure 1A), we set a threshold of five errors (or fewer) as a learning criterion. All assays were video-recorded for offline analysis (Logitech Webcam).

### Electrophysiology

**Figure 4:**
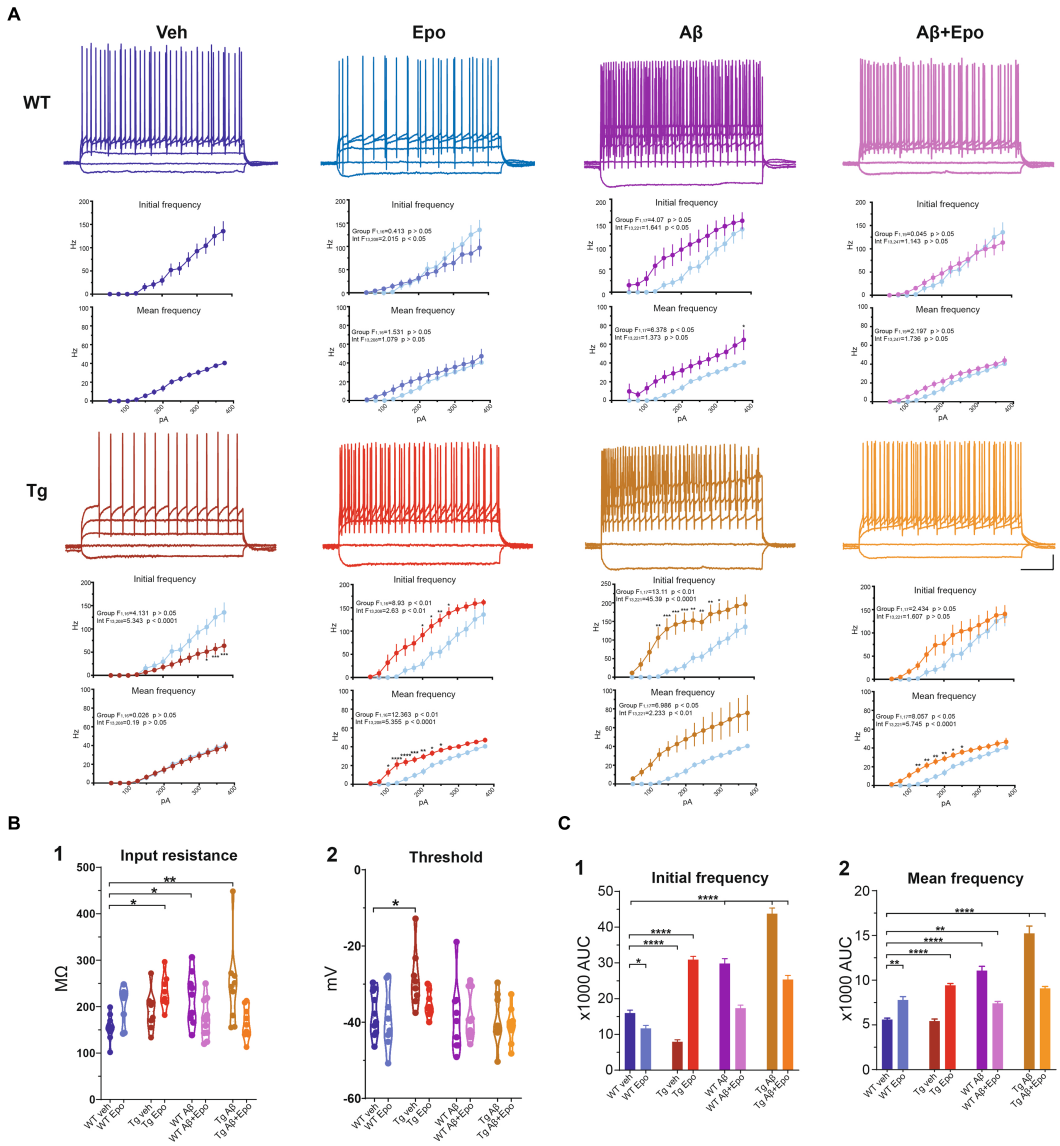
Intrinsic excitability of CA1 pyramidal neurons. **(A)** Representative voltage responses to 1 s long, square pulses of the following magnitudes: −100, 0, 100, 200 and 300 pA, for all experimental groups. Scale bars: 200 ms, 20 mV. I-F curves of the AP initial frequency and AP average frequency are included in each case. The control group was included in the other graphs (light blue) for comparison. **(B)** Quantification of **(1)** cell membrane input resistance and **(2)** threshold potential. **(C)** Quantification of the area under the curve (AUC) of the AP initial frequency **(1)** and AP average frequency **(2)** for all groups. WT = wild-type mice, Epo = Epothilone-D, Aβ = amyloid-β, Tg = transgenic mice, veh = vehicle. Asterisks denote significant differences as follows: *p < 0.05, **p < 0.01, ****p < 0.0001.

**Figure 5:**
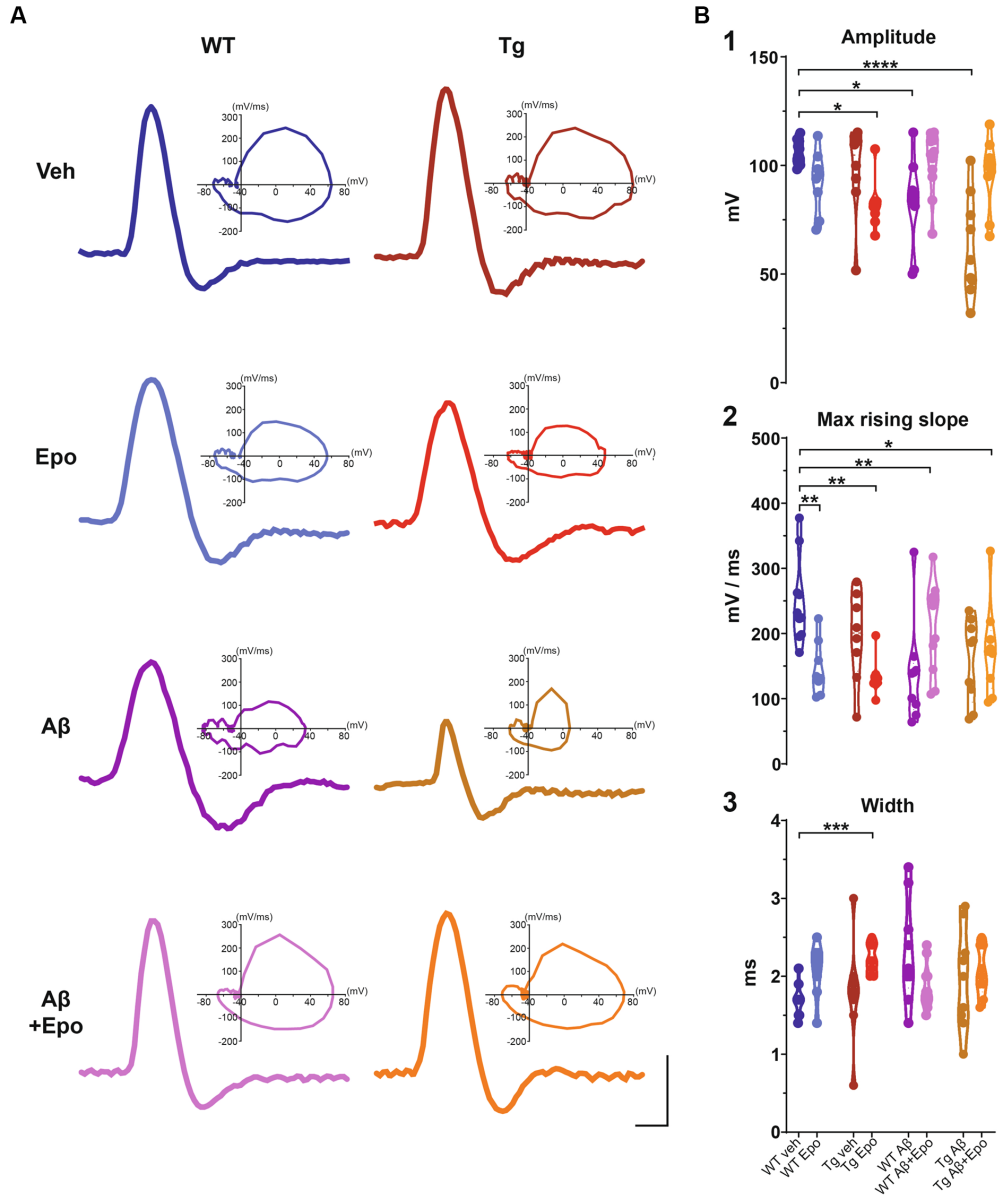
Action potential waveform. **(A)** Representative action potentials, obtained from the I/F curves elicited in CA1 pyramidal neurons of all experimental groups. The corresponding phase plot of each action potential is shown on the right. Scale bars: 1 ms, 50 mV. **(B)** Quantification of the amplitude **(1)**, maximum rising slope **(2)**, and width **(3)** of the action potentials of all experimental groups. WT = wild-type mice, Epo = Epothilone-D, Aβ = amyloid-β, Tg = transgenic mice, veh = vehicle. Asterisks denote significant differences as follows: *p < 0.05, **p < 0.01, ***p < 0.001, ****p < 0.0001.

**Figure 6:**
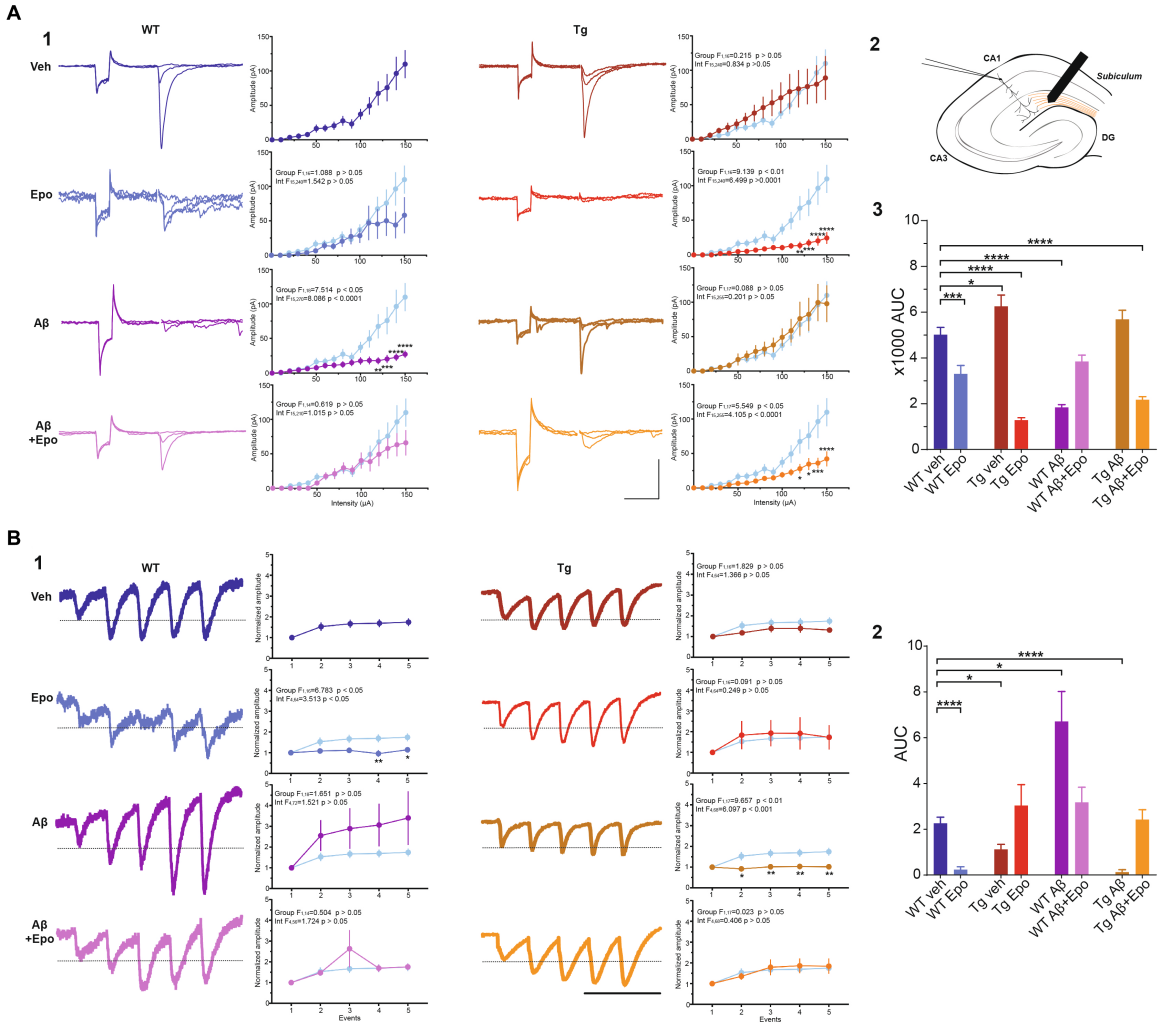
Transmission in the temporoammonic pathway. **(A)** EPSCs recorded in CA1 neurons **(1)** upon extracellular stimulation of the temporoammonic fibers at the *stratum lacunosum-moleculare* (schematized in **2**). For each experimental group there are three representative current traces that correspond to three stimulation intensities: 40, 60, and 100 μA. Scale bars: 100 ms, 100 pA. The quantifications of the input–output (I/O) curves are shown on the right. Pulse intensity ranges from 0 to 150 μA. **(3)** Area under the I/O curves shown in **(1)**. **(B)** EPSCs evoked by 25 Hz stimulation trains **(1)**. The representative current traces are the average of eight responses. The dotted line indicates the normalized amplitude of S1. Scale bar: 100 ms. Quantification of the mean normalized amplitudes (S1 set as 1) of the five evoked responses (S1-S5) are shown on the right. For the two panels, the control group was included in the graphs (light blue) for comparison. **(2)** Quantification of the area under the short-term plasticity (STP) curves with the dotted line in **(1)** set as baseline. WT = wild-type mice, Epo = Epothilone-D, Aβ = amyloid-β, Tg = transgenic mice, veh = vehicle, AUC = area under the curve. Asterisks denote significant differences as follows: *p < 0.05, ***p < 0.001, ****p < 0.0001.

After completing the behavioral tests, brain slices were obtained from each mouse for patch-clamp recordings (Figures 1A, 4–6). To do so, mice were anesthetized with pentobarbital (100 mg/kg; Adaya-Villanueva et al., 2010). Next, they were transcardially perfused with a cold low-sodium (substituted with sucrose) saline containing (in mM): 75 sucrose, 25 glucose, 25 NaHCO_3_, 73 NaCl, 2.5 KCl, 7 MgCl_2_, 0.5 CaCl_2_ (pH 7.4; 4°C) bubbled with carbogen (95% O_2_, 5% CO_2_). Mice were subsequently decapitated, and their brains were carefully dissected and submerged in ice-cold low-sodium saline. The two hemispheres were separated and glued horizontally at an angle of ~10°, onto a vibratome (HM 650 V, Thermo Scientific, USA) to obtain 350 μm thick horizontal slices with extensive hippocampal/entorhinal cortex connectivity (Méndez-Salcido et al., 2022). The slices were left to recover in a low-calcium/high-magnesium saline containing (in mM): 10 glucose, 25 NaHCO_3_, 125 NaCl, 2.5 KCl, 6 MgCl_2_, and 0.5 CaCl_2_ at 36°C. After at least 1 h of recovery, one slice was transferred to a recording chamber, located under a microscope (E-600FN Nikon) provided with differential interference contrast illumination and coupled to a perfusion system with a 3 mL/min flow of bubbled artificial cerebrospinal fluid (aCSF) containing (in mM): 10 glucose, 25 NaHCO_3_, 1.25 Na_2_HPO_4_, 125 NaCl, 2.5 KCl, 2 MgCl_2_, 2 CaCl_2_ at 34°C. Borosilicate glass capillaries (outside diameter: 1.5 mm; inside diameter: 0.86 mm; Sutter Instrument) were pulled (Flaming-Brown P-97, Sutter Instrument) to obtain 4–7 MΩ electrodes that were filled with an internal recording solution containing (in mM): 120 potassium gluconate, 20 KCl, 0.5 MgCl_2_, 10 HEPES, 0.5 EGTA, 2 NaATP, 0.3 NaGTP, pH = 7.3 adjusted with KOH 1 M. Patch sealing was made in voltage clamp mode, pipette capacitive currents were canceled in the cell-attached configuration, and series resistance was measured and compensated by 80% in the whole-cell configuration. Identification of hippocampal CA1 pyramidal neurons was based on their laminar location at the *stratum pyramidale*, their somatic morphology, and their intrinsic electrical properties such as low input resistance (100–250 MΩ), a time constant of 10–20 ms and evoked regular spiking with a frequency of 5–30 Hz after stimulation under 200 pA in current clamp mode (Balleza-Tapia et al., 2010; Peña et al., 2010; Tamagnini et al., 2015). Once we obtained a stable recording of a CA1 pyramidal neuron in current-clamp mode, current–voltage (I/V) and current-frequency (I/F) curves were obtained with square current pulses of 1 s, from −200 to 100 pA for I/V curves, and 0 to 375 pA for I/F curves, in steps of 25 pA. Subsequently, in voltage-clamp mode, spontaneous postsynaptic currents (sPSCs) were recorded for 5 min at a holding potential of −70 mV (Balleza-Tapia et al., 2010; Méndez-Salcido et al., 2022). To assess the synaptic properties of the temporoammonic (TA) innervation to the recorded CA1 pyramidal neurons, an extracellular stimulating concentric bipolar electrode was positioned on these fibers in the *stratum lacunosum-moleculare* of the subiculum (Arrigoni and Greene, 2004). Input–output curves (I/O) were obtained by evoking excitatory postsynaptic currents (EPSCs) with 100 μs square pulses of ascending amplitude (0 to 150 μA in steps of 10 μA), delivered at 0.05 Hz (Salgado-Puga et al., 2017; Alcantara-Gonzalez et al., 2019). Short-term plasticity (STP) was evaluated by applying trains of five pulses at 25 Hz and at the intensity that evoked an initial EPSC with an amplitude 40–50% of maximal response obtained in each individual I/O curve. Trains were delivered at 0.05 Hz (Flores-Martínez and Peña-Ortega, 2017; Torres-Flores and Peña-Ortega, 2022). During voltage-clamp recordings, series resistance was monitored with 20 ms-long and −2 mV commands applied 40 ms before the extracellular stimulation used to induce the EPSCs. Cells that displayed series resistance changes above 20% were discarded. Signals were amplified, low-pass filtered at 5 kHz (Axopatch 200b, Molecular Devices), and digitized at 10 kHz (Digidata, Molecular Devices).

### Nissl staining

**Figure 7:**
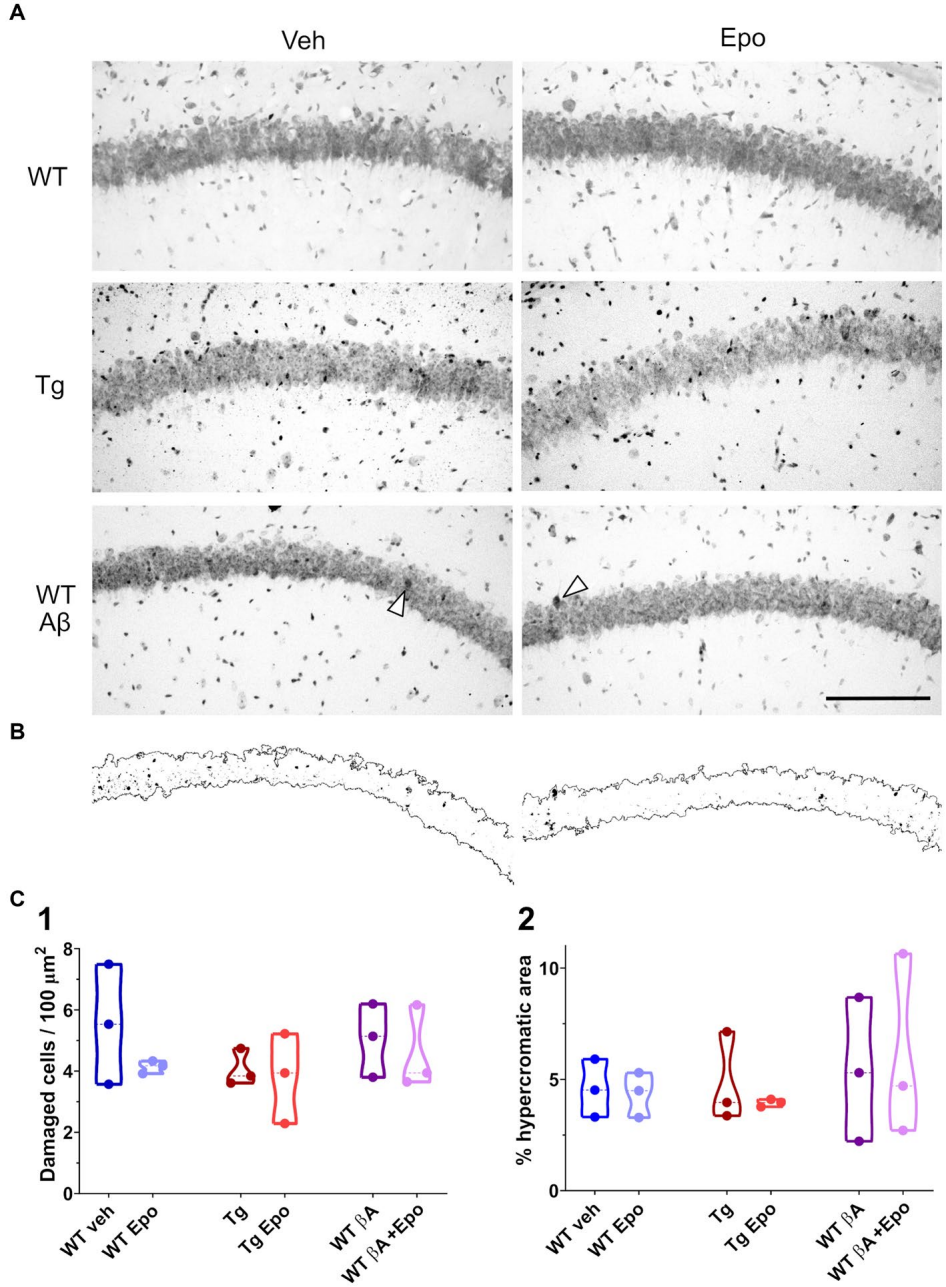
Hippocampal CA1 integrity. **(A)** Representative micrographs of Nissl-stained hippocampal sections obtained from animals of the indicated groups. Scale bar: 150 μm (also applies for **B**). **(B)** Binarized CA1 area extracted from the micrographs at the bottom of panel **(A)** as well as the hyperchromic areas that surpassed an internal threshold determined by the arrowhead in each micrograph. **(C)** Quantification of the number of damaged cells (**1**; i.e. hyperchromic and/or irregular cells) in a given area of the CA1 pyramidal cell layer and of the proportion of hyperchromic area from the same area in binarized images **(2)**. WT = wild-type mice, Epo = Epothilone-D, Aβ = amyloid-β, Tg = transgenic mice, veh = vehicle.

In an independent group of mice, which were not behaviorally tested, CA1 integrity was evaluated in Nissl-stained sections (Peña and Tapia, 1999; Pena and Tapia, 2000; SantaCruz et al., 2005; Zussy et al., 2013; Eslamizade et al., 2015; Salgado-Puga and Pena-Ortega, 2015; Sharma et al., 2016; Raj et al., 2019). Briefly, animals were anesthetized with pentobarbital, transcardially perfused with low-sodium saline, and the brains were extracted and fixed overnight in 4% paraformaldehyde (Sigma, USA) in phosphate-buffered saline (PBS; Sigma, USA), and then in a sucrose solution (30% in PBS) for 3 days. Cryosections (30 μm thick) were obtained (CM 350S, Leica, Wetzlar, Germany) at the level of the dorsal hippocampus, mounted on gelatinized slides, stained with 0.1% cresyl violet (Sigma, USA) and coverslipped with Entellan medium (Sigma, USA). Observations were conducted by bright field microscopy (Axioplan 2, Zeiss, Germany; Figure 7A). CA1 hyperchromic and irregular cells (damaged cells) were counted using Image-J software (Peña and Tapia, 1999; Pena and Tapia, 2000). Additionally, also with Image-J, we calculated the proportion of CA1 hyperchromic area by automatically delimiting the CA1 pyramidal layer and subsequently used a typical hyperchromic cell or a condensed nucleus within any given micrograph to set an individual hyperchromic threshold. Then, we quantified all the area above this threshold as a proportion of the whole captured CA1 area (Figure 7B). For this purpose, digitized images acquired using a ×20 objective and the software Zen (Zeiss, Germany) were brought to the automatic level of contrast set by using Image-J. Three slides were analyzed for each mouse. Cells in the region of interest were calculated per mouse and averaged per group.

### Data analysis

Video recordings were analyzed offline with the Matlab Optimouse toolbox (Ben-Shaul, 2017). Total distance, averaged velocity, and errors were measured for each assay in the HW test. Classical encoding and retrieval indexes (EI_D1_ and RI_D1-D2_) were calculated with the number of errors observed when mice solved the #4 HW maze (Rogers and Kesner, 2003; Lee and Kesner, 2004; Jerman et al., 2006; Vago et al., 2007; Hunsaker et al., 2008; Churchwell et al., 2010; Méndez-Salcido et al., 2022; Figure 3C_1,2_). We also calculated encoding indexes for D2 and D3 (EI_D2_ and EI_D3_; Supplementary Figure 1A_1,2_) as well an overall encoding index from the beginning of D1 to the end of D3 (EI_D1-D3_; Supplementary Figure 1A_3_). A flexibility index (FI; Supplementary Figure 1B) and a re-encoding index (REI; Supplementary Figure 1C) were also calculated with the number of errors observed when mice solved the #9 HW maze. Indexes were calculated as follows:


$$EI_{D1}=Err_{D1-I}-Err_{D1-F}EI_{D2}=Err_{D2-I}-Err_{D2-F}EI_{D3}=Err_{D3-I}-Err_{D3-F}$$

$$RI_{D1-D2}=Err_{D1-F}-Err_{D2-I}RI_{D2-D3}=Err_{D2-F}-Err_{D3-I}FI=Err_{D3-F}-Err_{D4-I}$$

$$\begin{array}{l} REI=Err_{D4-I}-Err_{D4-F}\;\\ EI_{D1-D3}=Err_{D1-I}-Err_{D3-F} \end{array}$$

Where:

$Err_{Dn-I}$ is the averaged error of the first 5 trials on day *n*

$Err_{Dn-F}$ is the averaged error of the last 5 trials on day *n*

Electrophysiological recordings were analyzed using custom-made Matlab scripts and Clampfit v.10 (Molecular Devices; see Figure 1B_1-3_). Membrane resting potential was measured in current clamp at zero holding current just after whole-cell configuration was achieved. The membrane input resistance was measured as the slope of the I/V curve from 0 to 100 pA stimulation values. Membrane time constant (τ) was calculated from a simple exponential fit of the ascending voltage response to a +25 pA square pulse. Sag rectification was obtained as the quotient of the initial peak voltage response to a −200 pA hyperpolarizing step current divided by the voltage at the end of the response. Rheobase was determined as the minimal current value that evoked at least one action potential (AP). Threshold potential was considered as the first membrane potential value with a derivative value above 20 mV/ms (Hatch et al., 2017). All AP properties were measured from the first AP evoked in the I/F curve. AP maximal rise slope was considered as the highest derivative value of the rising phase voltage (Colbert et al., 1997). AP amplitude was measured from threshold to peak voltage (Figure 1B_2_). AP width was measured as the time from the beginning of the AP to the point where the voltage reached the threshold potential (Figure 1B_2_). This same time point was considered as the onset of the afterhyperpolarization (AHP; Figure 1B_2_). AHP amplitude was calculated from its onset to its most negative peak. AHP duration was measured from its onset to the time when voltage depolarization stopped (slope = 0 mV/ms). Initial firing rate was calculated as the instantaneous firing frequency from the first pair of APs in each train, whereas mean firing rate was calculated as the average of the instantaneous frequencies of all pairs of APs in each train (Figure 1B_1_). The mean area under the I/F, I/O and STP curves was also calculated. The amplitude of the EPSCs was measured from baseline to its peak current (Figure 1B_3_). STP curves were normalized to the amplitude of the first EPSC (S1; set as one; Figure 1B_3_). Thus, the area under the STP curve was calculated considering a baseline value of one. The detection threshold for sPSCs was set at 3.5 times the standard deviation of the whole signal. All events were visually inspected to confirm normal sPSC waveform (i.e., a linear rising phase and an exponential decaying phase; Balleza-Tapia et al., 2010).

All data are presented as mean ± standard error of the mean (SEM). The n in behavioral experiments represents the number of animals, whereas in electrophysiological measurements it represents the number of neurons. A Shapiro-Wilcoxon normality test was applied to all data sets. Repeated measures analysis of variance (ANOVA) with Dunnett’s post-hoc test was used to evaluate significant differences among means. Welch’s ANOVA with Dunnett’s T3 post-hoc test was applied if variances among groups were significantly different. For I/F, I/O, and STP curves, two-way ANOVA with Sidak’s post-hoc test was used. Comparisons in the post-hoc tests were made with respect to the WT group administered with vehicle. Significance was defined as α = 0.05.

## Results

### Epo-D impairs locomotion in Tg mice

Previous reports of MSA side-effects indicate that these drugs impair locomotion and could thus affect spatial navigation performance (Ray et al., 2011). Therefore, we analyzed the movement and exploration of all mice in an open field after the first administration of either Epo-D or its vehicle (Figure 2). In the WT groups, Epo-D did not change the total traveled distance in the open field (Figure 2B_1_). In contrast, administration of Epo-D to Tg mice diminished their total traveled distance (ANOVA, F_7,56_ = 8.425, *p* < 0.0001, Dunnett’s post-hoc test; distance_Tg veh_ = 40.308 ± 2.562 m, adjusted *p* < 0.01; distance_Tg Epo_ = 24.671 ± 2.451 m adjusted *p* < 0.01; Figure 2B_1_). Similar effects of Epo-D (decrease in traveled distance) were observed in Tg mice with global Aβ (distance_Tg Aβ + Epo_ = 26.298 ± 2.104 m, adjusted *p* < 0.05). We did not find changes in the proportion of time spent in the center/periphery evaluated through the center index (CI) in any of the groups (Welch’s ANOVA test, F_7,23.05_ = 3.379, adjusted *p* > 0.05, Dunnett’s T3 post-hoc test; Figure 2B_2_).

### Aβ- and P-tau induce mild alterations in spatial memory retrieval

After the open field test, we evaluated the mice’s spatial learning and memory with the Hebb-Williams test (Méndez-Salcido et al., 2022; Figures 1A, 3B; Supplementary Figure 1). All animals except Tg mice with global Aβ had an average of five or fewer errors (learning threshold) by the end of day one (D1-F) and maintained their performance below threshold until D3 (Figures 3A,C). We found no difference in the learning curve of WT animals with global Aβ compared to control mice (group factor F_1,12_ = 1.273, *p* > 0.05; interaction F_7,84_ = 1.321, *p* > 0.05; Figure 3A), but we did identify a significant difference between the learning curves of Tg mice and control mice (group factor F_1,14_ = 6.204, *p* < 0.05; interaction F_7,98_ = 1.753, *p* > 0.05; Figure 3A). Despite this difference, all groups reached learning levels that were not significantly different from those of control animals after repeated training for three consecutive days (Supplementary Figure 1C). As mentioned, we quantified the classical encoding and retrieval indexes (EI_D1_ and RI_D1-D2_, respectively; see Methods; Figure 3C_1,2_; Lee and Kesner, 2004; Jerman et al., 2006; Churchwell et al., 2010; Méndez-Salcido et al., 2022) and found no differences in the classical EI_D1_ (i.e., during the first training day) among groups (ANOVA F_7,54_ = 1.032, *p* > 0.05, Dunnett’s post-hoc test; Figure 3C_1_). No differences among groups were found for EI_D2_ (Welch’s ANOVA F_7,22.653_ = 1.413, *p* > 0.05, Dunnett’s T3 post-hoc test) or EI_D3_ (Welch’s ANOVA F_7,21.83_ = 2.018, *p* > 0.05, Dunnett’s T3 post-hoc test; Supplementary Figure 1A). However, classical retrieval index (RI_D1-D2_), which evaluates retrieval of the information gathered on D1 (RI_D1-D2_), was reduced in WT mice with global Aβ (F_7,54_ = 2.466, *p* > 0.05, Dunnett’s post-hoc test; RI_D1-D2; WT veh_ = 3.438 ± 0.325; RI_D1-D2; WT Aβ_ = −0.629 ± 1.086) and in Tg mice microinjected with vehicle (RI_D1-D2; Tg veh_ = −1.167 ± 0.931; Figure 3C_2_) compared to WT mice microinjected with vehicle. This abnormal RI_D1-D2_ was also reflected as a positive slope (arrows in Figure 3A) in the performance curve of the animals between the end of D1 and the beginning of D2 (i.e., more errors at the beginning of D2 than at the end of D1; Figure 3A). At the beginning of D3, we calculated the RI_D2-D3_ (retrieval of the information gathered during D1 and D2). Tg mice exhibited a higher RI_D2-D3_ than the rest of the groups (Welch’s ANOVA F_7,22.38_ = 2.359, *p* = 0.058, Dunnett’s T3 post-hoc test, adjusted *p* < 0.05; Supplementary Figure 1B), mainly because only this group had a significantly high number of errors at the end of D2 (Figure 3A) and on D3 they made fewer errors (Figure 3A).

### The combination of Aβ and P-tau pathology affects cognitive flexibility

Since Aβ and P-tau had similar detrimental effects on retrieval, we expected their combination to potentiate their individual pathological effects (Ittner et al., 2010; Zempel and Mandelkow, 2012). Unexpectedly, Tg mice with global Aβ had a RI_D2-D3_ similar to that of WT mice (RI_D2-D3; Tg Aβ_ = 1.601 ± 1.736; Figure 3C_2_). However, Tg mice with global Aβ exhibited a poor performance on D1 and D2, as their mean errors did not reach the 5-error learning threshold, which was only achieved during the last 5 trials of D2 (Figure 3A). Despite that Tg mice with global Aβ did not exhibit more errors at the beginning of D2 than at the end of D1 (i.e., they had a normal RI_D2-D3_; Figure 3C_2_), they did sustain a high number of errors (Figure 3A_2_). It is important to stress that the high variability of errors seen during the retrieval phase (end of D1 and beginning of D2) in this group of mice precluded a significant change in RI_D2-D3_ (Figure 3C_2_).

### Epo-D rescues Aβ- and P-tau-induced impairments of retrieval

As hypothesized, Epo-D treatment in WT mice with global Aβ (RI_D2-D3_ = 1.571 ± 0.785; Figure 3C_2_) and Tg mice (RI_D2-D3_ = 1.578 ± 0.642; Figures 3A_2_,C_2_) allowed them to retrieve information as control animals did. Moreover, Epo-D reduced the high error levels exhibited by Tg mice with global Aβ (Figure 3A). In fact, Tg mice with global Aβ treated with Epo-D reached the learning threshold (less than 5 errors) by the end of D1, as seen in the rest of the groups (Figure 3A). As expected, Tg mice injected with Aβ and Epo-D exhibited a RI similar to the one shown by the WT group (RI_D2-D3_ = 1.300 ± 0.398; Figure 3C_2_).

### Epo-D reduces cognitive flexibility in WT mice

We also evaluated the effects of Aβ, P-tau, their combination, and Epo-D on the cognitive flexibility (i.e., reversal learning) required to solve the HW test when the maze is changed (Torres-Flores and Peña-Ortega, 2022). As expected, all mice made more mistakes when completing a new maze (D4-I; Figure 3A) but improved their performance upon repetition (D4-F; Figure 3A), thus showing similar REIs (Welch’s ANOVA F_7,22.574_ = 1.940, *p* > 0.05, Dunnett’s T3 post-hoc test; Supplementary Figure 1C). All experimental groups, except for WT animals with global Aβ or Tg mice treated with vehicle, exhibited significantly more errors than control animals when initially encountered with the new maze (D4-I; Figure 3A). To further evaluate the ability of mice to recognize the change in the maze and adjust their behavior, we measured the FI (Figure 3C_3_). Surprisingly, the FI was more negative in WT mice that received Epo-D (Welch’s ANOVA with F_7,22.93_ = 3.389, *p* < 0.01, Dunnett’s T3 post-hoc test; FI_WT veh_ = −3.486 ± 0.6337; FI_WT Epo_ = −7.422 ± 0.9829; Figure 3C_3_) or animals that received Epo-D and were microinjected with Aβ (FI_WT Aβ + Epo_ = −6.743 ± 0.508; Figure 3C_3_). This deleterious effect of Epo-D on FI was not observed in Tg mice (FI_Tg_ = −5.600 ± 1.628; Figure 3C_3_), which were similar to control animals (adjusted *p* = 0.712). Although the FI of Tg mice was not significantly different from that of control animals, some Tg animals exhibited a positive FI (Figure 3C_3_), which was partially due to the elevated number of errors that these mice presented at the end of the third training day in the initial maze (Figure 3A). Despite the alterations in cognitive flexibility observed in some groups (Figures 3A,C_3_), most animals reduced the number of errors by the end of the same day exposed to the new maze, which is reflected in similar REIs (Supplementary Figure 1C).

### Aβ and P-tau have divergent effects on CA1 neuron excitability

Retrieval in the HW maze is highly dependent on CA1 and on its inputs from the entorhinal cortex through the TA pathway (Jerman et al., 2006; Churchwell et al., 2010). Thus, we analyzed the intrinsic and synaptic properties of CA1 principal neurons in the presence of Aβ, P-tau, their combination, and Epo-D (Figure 1B). Recordings were performed in slices obtained after animals had completed behavioral tests (Figure 1A).

### Aβ increases CA1 intrinsic excitability

When we analyzed the intrinsic properties of CA1 neurons in WT mice treated with Aβ, we found an increase in excitability (Figure 4A) that correlated with higher input resistance (ANOVA, F_7,64_ = 3.808, *p* < 0.01, Dunnett’s post-hoc test; Rm_WT veh_ = 155.401 ± 8.361 MΩ; Rm_WT Aβ_ = 218.222 ± 18.442 MΩ; Figure 4B_1_). The Aβ-induced increase in neuronal excitability is reflected as an increase in mean firing frequency, measured both at the beginning (two-way ANOVA, interaction F_91, 221_ = 1.641, *p* < 0.05, Sidak’s post-hoc test; Figure 4A), and during the train (two-way ANOVA, group F_1, 17_ = 6.378, *p* < 0.05, Sidak’s post-hoc test; Figure 4A), as well as in the area under the I/F curves (initial I/F area: ANOVA, F_7,64_ = 119.738, *p* < 0.0001, Dunnett’s post-hoc test, AUC_WT veh_ = 15971.054 ± 2573.957; AUC_WT Aβ_ = 29,048 ± 3746.774; mean I/F area: ANOVA, F_7,64_ = 65.378, *p* < 0.0001, Dunnett’s post-hoc test; AUC_WT veh_ = 5597.638 ± 496.226, AUC_WT Aβ_ = 11275.707 ± 1461.971; Figure 4C_1,2_). Aβ also changed the AP waveforms (Figure 5) by reducing their amplitude (_WT veh_ = 106.289 ± 1.762 mV, _WT Aβ_ = 82.244 ± 6.867 mV; Figure 5B_1_) and their rising slope (_WT veh_ = 248.501 ± 20.706 mV/ms, _WT Aβ_ = 138.177 ± 26.017 mV/ms; Figure 5B_2_; Table 2).

**Table 2:** Summary of electrophysiological properties of CA1 principal neurons and TA pathway. (W), Welch’s ANOVA test.

	WT veh	WT Epo	WT A**β**	WT A**β** + Epo	Tg veh	Tg Epo	Tg A**β**	Tg A**β** + Epo	ANOVA	
	*n* = 10	*n* = 8	*n* = 11	*n* = 10	*n* = 8	*n* = 8	*n* = 9	*n* = 9	*F*	*p*
Passive properties										
Vm (mV)	−59.456 ± 1.875	−56.118 ± 2.497	−58.279 ± 2.326	−63.701 ± 1.974	−53.388 ± 2.537	−63.538 ± 2.377	−63.589 ± 1.191	−65.259 ± 2.596	3.662	<0.01
Tau (ms)	15.037 ± 2.987	10.419 ± 2.098	9.137 ± 1.325	12.594 ± 2.087	15.357 ± 1.661	19.086 ± 1.178	17.241 ± 1.348	19.411 ± 2.199	3.439	<0.01
Rm (MΩ)	155.401 ± 8.361	202.737 ± 14.593	218.222 ± 18.442*	172.809 ± 12.381	191.275 ± 15.051	231.963 ± 12.555*	245.011 ± 29.531**	164.955 ± 11.313	4.063	<0.01
Cm (pF)	75.453 ± 6.701	50.845 ± 9.921	45.621 ± 7.966	77.339 ± 14.351	82.066 ± 8.503	84.034 ± 6.797	77.395 ± 9.996	128.711 ± 23.884*	3.887	<0.01
Sag (proportion)	0.061 ± 0.016	0.094 ± 0.029	0.042 ± 0.009	0.047 ± 0.004	0.171 ± 0.011***	0.223 ± 0.011****	0.033 ± 0.006	0.036 ± 0.006	(W) 51.801	<0.0001
Active properties										
Rheobase (pA)	175.000 ± 17.078	125.000 ± 18.898	111.111 ± 19.593*	132.364 ± 17.309	168.750 ± 16.870	96.875 ± 11.017**	75.000 ± 11.785***	94.444 ± 8.098**	5.104	<0.01
Threshold (mV)	−37.772 ± 1.839	−39.085 ± 2.849	−38.892 ± 3.066	−38.674 ± 1.787	−28.649 ± 2.736*	−35.324 ± 1.193	−39.572 ± 2.114	−40.782 ± 1.522	2.813	<0.05
AP amplitude (mV)	106.289 ± 1.762	92.263 ± 5.121	82.244 ± 6.867*	101.142 ± 4.317	97.825 ± 7.444	82.136 ± 4.095*	62.756 ± 7.727****	95.98 ± 5.5	6.488	<0.0001
AP rise time (ms)	0.820 ± 0.061	1.050 ± 0.070	1.022 ± 0.76	0.836 ± 0.047	0.875 ± 0.062	1.025 ± 0.041	0.867 ± 0.093	0.944 ± 0.058	2.018	>0.05
AP rise slope (mV/ms)	248.500 ± 20.706	146.299 ± 14.773**	138.177 ± 26.017**	211.265 ± 20.704	194.549 ± 24.379	133.514 ± 10.014**	158.115 ± 21.131*	175.646 ± 23.278	3.767	< 0.01
Width (ms)	1.690 ± 0.064	2.075 ± 0.122	2.311 ± 0.221	1.864 ± 0.086	1.788 ± 0.233	2.225 ± 0.067***	1.967 ± 0.215	2.056 ± 0.107	(W) 4.869	<0.001
Half-width (ms)	0.970 ± 0.052	1.225 ± 0.070	1.311 ± 0.119	1.073 ± 0.054	1.138 ± 0.094	1.325 ± 0.045***	1.133 ± 0.128	1.222 ± 0.619*	(W) 4.074	<0.01
AHP amplitude (mV)	30.970 ± 3.165	36.000 ± 2.620	25.400 ± 4.033	27.923 ± 1.911	34.487 ± 3.028	25.875 ± 1.519	21.711 ± 2.169	28.579 ± 2.659	2.846	<0.05
AHP width (ms)	2.222 ± 0.205	2.388 ± 0.194	3.089 ± 0.516	2.245 ± 0.101	2.538 ± 0.205	2.913 ± 0.168	2.622 ± 0.220	2.459 ± 0.108	1.619	>0.05
Initial I/F area (AUC)	15971.054 ± 813.654	11678.774 ± 817.663*	29048.585 ± 1344.48****	17343.250 ± 839.945	7942.386 ± 553.382****	30907.319 ± 904.672****	43757.338 ± 1585.600****	25344.144 ± 1160.367****	120.777	<0.0001
Mean I/F area (AUC)	5597.638 ± 156.922	7777.248 ± 381.555**	11070.220 ± 473.830****	7404.316 ± 209.147**	5419.564 ± 234.215	9416.027 ± 207.946****	15231.681 ± 819.967****	9082.273 ± 209.873****	66.796	<0.0001
I/O area (AUC)	5019.101 ± 313.804	3306.011 ± 367.342***	1838.851 ± 121.273****	3846.083 ± 277.119	6255.751 ± 491.793*	1283.875 ± 105.642****	568.2224 ± 400.667	2175.556 ± 132.800****	38.007	<0.0001
STP area (AUC)	2.265 ± 0.263	0.241 ± 0.122****	6.709 ± 1.309*	3.176 ± 0.661	1.122 ± 0.218*	3.037 ± 0.911	0.052 ± 0.108****	2.424 ± 0.429	(W) 16.859	<0.0001

The *n* represents the number of neurons used for quantifications. *p < 0.05, **p < 0.01, ***p < 0.001, ****p < 0.0001.

### Tau pathology reduces intrinsic excitability

In agreement with the reported hypoexcitable profile of CA1 principal neurons in Tg mice (Hatch et al., 2017), we found that CA1 neurons from Tg mice had a depolarized firing threshold potential (ANOVA, F_7,64_ = 2.813, *p* < 0.01, Dunnett’s post-hoc test; threshold_WT veh_ = −37.772 ± 1.839 mV; threshold_Tg veh_ = −28.649 ± 2.736 mV; Figure 4B_2_) that was related to a reduced initial firing frequency (initial I/F AUC = 7942.386 ± 1565.202; Figure 4A). We also found an increased sag potential in these neurons (Welch’s ANOVA, F_7,25.869_ = 51.801, *p* < 0.0001, Dunnett’s T3 post-hoc test; sag_WT veh_ = 0.061 ± 0.016; sag_Tg veh_ = 0.171 ± 0.011; Supplementary Figure 2), which has also been previously described (Booth et al., 2016b).

### Aβ-induced hyperexcitability overcomes P-tau-induced hypoexcitability

Since Aβ and P-tau pathology caused divergent effects on CA1 pyramidal neuron excitability (Figure 4), we evaluated their effects when combined and found that Tg mice with global Aβ exhibited increased firing rates (initial I/F AUC = 43757.338 ± 4757.842; mean I/F AUC = 15231.681 ± 2459.868; Figures 4A,C), combined with increased input resistance (245.011 ± 29.531 MΩ; Figure 4B_1_). In the presence of P-tau and Aβ, AP amplitude and waveform were also altered (amplitude = 62.756 ± 7.727 mV; maximum rising slope = 158.115 ± 21.131 mV/ms; Figure 5; Table 2). Unexpectedly, Aβ reduced the sag potential in CA1 Tg neurons (sag_Tg Aβ_ = 0.033 ± 0.006; Supplementary Figure 2) to similar levels as those of control neurons (sag_WT veh_ = 0.061 ± 0.016; Supplementary Figure 2).

### Epo-D by itself modifies AP waveform

Before analyzing its likely protective effects against Aβ- or P-tau-induced pathology, we evaluated the effects of Epo-D on CA1 neurons from healthy WT mice and found that neurons recorded from WT mice injected with Epo-D exhibited a mixed change in firing frequency since it increased the area under the mean I/F curve (7777.248 ± 1079.223; Figure 4C_2_), although Epo-D also reduced the initial frequency of the evoked trains of APs (11678.774 ± 3205.784; Figure 4C_1_). Epo-D also reduced the maximum rising slope of the AP (146.299 ± 14.773 mV/ms; Figure 5; Table 2).

### Epo-D partially rescues CA1 neuron excitability in the presence of Aβ, P-tau, and their combination, but distorts AP waveform in these conditions

Given that Epo-D rescued the memory deficits induced by Aβ, and P-tau, and their combination, we evaluated if it could also rescue normal excitability in CA1 principal neurons under these pathological conditions (Figure 4). When Epo-D was administered to WT mice with global Aβ, it restored their input resistance to a value not unlike that of the control group (172.521 ± 13.684 MΩ; Figure 4B_1_), while their firing frequency became normal when measured either at the beginning of the evoked train (initial I/F AUC = 18502.531 ± 2793.805; Figure 4C_1_) or during the whole train (mean I/F AUC = 7677.738 ± 691.251; Figure 4C_2_). In Tg neurons, Epo-D shifted the firing threshold potential towards a normal value (−35.324 ± 1.193 mV; Figure 4B_2_), increased their Rm (231.963 ± 12.555 MΩ; Figure 4B_1_), and robustly increased the firing rate (initial I/F AUC = 30907.319 ± 2558.783; mean I/F AUC = 9416.027 ± 588.163; Figure 4C). However, Epo-D altered the AP waveform of Tg neurons as compared to the AP waveform of WT neurons (Figure 5; Table 2). In WT neurons, Epo-D only reduced the AP rising slope (Figure 5B_2_), whereas in Tg neurons it reduced the AP rising slope (133.514 ± 10.014 mV/ms; Figure 5B_2_) and amplitude (82.138 ± 4.095 mV; Figure 5B_1_) whereas it increased the AP width (Tg Epo = 2.225 ± 0.067 ms; Figure 5B_3_). Epo-D partially rescued the firing rate of neurons recorded from Tg animals with global Aβ (initial I/F AUC = 25344.144 ± 3481.106; mean I/F AUC = 9082.273 ± 629.619; Figure 4C). Furthermore, in these animals, Epo-D normalized their threshold potential (−40.782 ± 1.522 mV; Figure 4B_2_), input resistance (164.955 ± 11.313 MΩ; Figure 4B_1_), and AP waveform (maximum rising slope = 175.646 ± 23.278 mV/ms; Figure 5; Table 2).

### Global synaptic input to CA1 neurons is increased by the combination of Aβ and Epo-D

We analyzed the global synaptic input to CA1 pyramidal neurons by measuring the amplitude and frequency of their sPSCs (see Methods; Supplementary Figure 3) and found no difference in such parameters in most of the experimental groups (ANOVA, F_7,60_ = 0.856, *p* > 0.05; Dunnett’s post-hoc test; Supplementary Figure 3B_1_). Only the group of WT animals treated with both Aβ and Epo-D exhibited an increase in sPSC frequency (ANOVA, F_7,60_ = 3.892, *p* < 0.01, Dunnett’s post-hoc test; frequency_WT veh_ = 2.566 ± 0.384 Hz, frequency_WT Aβ + Epo_ = 4.873 ± 0.401 Hz; Supplementary Figure 3B_2_).

### Aβ and P-tau have divergent effects on the temporoammonic pathway

TA pathway ablation has been related to impaired consolidation and retrieval in the HW test (Vago et al., 2007). Since consolidation and retrieval were affected by Aβ and P-tau, we examined if these pathological conditions, and Epo-D treatment influenced the TA pathway. We found that Aβ substantially reduced the amplitude of EPSCs evoked by different stimulation intensities at the TA pathway (two-way ANOVA, interaction F_1,18_ = 7.5147.514, *p* < 0.05, Sidak’s post-hoc test; Figure 6A_1_), which is also observed in the area under the I/O curve (ANOVA, F_7,60_ = 38.007, *p* < 0.0001, Dunnett’s post-hoc test; AUC_WT veh_ = 5019.101 ± 992.336; AUC_WT Aβ_ = 1838.851 ± 383.456; Figure 6A_3_). Aβ also changed the STP, as reflected in the area under the STP curve (Welch’s ANOVA, F_7,23.754_ = 18.376, *p* < 0.0001, Dunnett’s T3 post-hoc test; AUC_WT veh_ = 2.265 ± 0.263; AUC_WT Aβ_ = 6.709 ± 1.309; Figure 6B_2_).

The EPSCs recorded from Tg mice tended to have higher amplitudes when the TA pathway was stimulated at mild intensities (n. s., adjusted *p* = 0.275; 60–100 μA intensities; Figure 6A_1_). This increase in the amplitude of TA-induced EPSCs in Tg mice reached significance when the area under the I/O curve was measured (AUC = 6255.751 ± 1390.588; adjusted *p* = 0.027; Figure 6A_3_). TA synapses in Tg mice also produced less facilitation during the 25 Hz stimulation train (AUC = 1.122 ± 0.218; Figure 6B_2_).

When Aβ and P-tau pathology were combined, the amplitudes of EPSCs evoked by stimulating the TA pathway at different intensities were unchanged (Figure 6A_1_), and the area under the I/O curve remained unaltered (AUC = 5684.222 ± 1202.389; adjusted *p* = 0.440). However, the EPSCs evoked during the 25 Hz train were similar among them (i.e., lack of facilitation; Figure 6B_1_). Thus, the area under the STP curve was smaller than that of the control group (AUC = 0.052 ± 0.092; adjusted *p* < 0.0001; Figure 6B_2_).

### Epo-D depresses the transmission of the TA pathway and changes its short-term plasticity

When evaluating whether Epo-D *per se* could influence synaptic transmission in the TA pathway, we found that in WT neurons, Epo-D tended to reduce the EPSC amplitudes at the higher tested intensities (two-way ANOVA, interaction F_15,240_ = 1.542, *p* = 0.091, Dunnett’s post-hoc test, adjusted *p* = 0.059; Figure 6A_1_), which was reflected in a reduction of the area under the I/O curve (AUC 3306.011 ± 1039.191; Figure 6A_3_). In the presence of Epo-D the EPSCs evoked during the 25 Hz train were similar among them (i.e., lack of facilitation; Figure 6B_1_), which was reflected in a small area under the STP curve (AUC = 0.241 ± 0.122; Figure 6B_2_).

### Epo-D rescued Aβ-induced alterations in the TA pathway

As expected, and in spite of its own effects on the TA pathway, Epo-D rescued EPSC amplitudes (Figure 6A_1_) and, therefore, the area under the I/O curve (AUC = 3846.083 ± 678.841; Figure 6A_3_) of TA transmission in CA1 neurons recorded from WT mice with global Aβ. The EPSCs evoked during the 25 Hz train were similar to those of control slices (Figure 6B_1_). Thus, the area under the STP curve of WT mice administered with Aβ and Epo-D also showed normal values (AUC = 3.176 ± 0.661; Figure 6B_2_).

### Epo-D depresses the transmission of the TA pathway in animals with tau pathology

Epo-D dramatically reduced synaptic transmission in CA1 neurons recorded from Tg mice (I/O curve: two-way ANOVA, group F_1,16_ = 9.139, *p* < 0.01, Sidak’s post-hoc test; AUC = 1283.875 ± 298.773; Figure 6A). This depressive effect of Epo-D was also observed in Tg mice with global Aβ (I/O curve: two-way ANOVA, group F_1,17_ = 5.549, *p* < 0.05, Sidak’s post-hoc test; AUC = 2175.556 ± 398.355; Figure 6A). The EPSCs evoked during the 25 Hz train showed normal facilitation upon Epo-D treatment in Tg mice (AUC = 3.211 ± 0.807) and Tg mice with global Aβ (AUC = 2.424 ± 0.429; Figure 6B_2_).

### Aβ, P-tau, and EpoD do not affect hippocampal CA1 integrity

Since Aβ, P-tau and EpoD have been associated with different aspects of neuronal damage (SantaCruz et al., 2005; Zussy et al., 2013; Eslamizade et al., 2015; Sharma et al., 2016; Raj et al., 2019), we evaluated CA1 integrity by cresyl violet staining (Figure 7A) and found that none of these conditions induced a change in the intensity of the Nissl staining (i.e., no increase in hyperchromic cells; Figure 7B) or in the number of damaged cells (Figure 7C).

## Discussion

By measuring hippocampal-dependent behavior and with patch-clamp recordings, we compared the pathological consequences of Aβ, P-tau, and their combination, while also testing the protective and deleterious effects of Epo-D, a microtubule stabilizer, in these conditions. We found that both Aβ and P-tau produced opposing, synergistic, and even antagonistic effects on neuronal excitability while inducing similar mild memory impairments. Epo-D prevented several alterations, but also it had negative effects on neuronal excitability and particular aspects of cognition that depended on preexisting physiological or pathological conditions. The experimental outputs of our study are certainly elaborate, but we think that it is crucial to compare the effects of Aβ and P-tau (as well as their combination) in similar conditions, and to test the effects of Epo-D under these same conditions (as well as under physiological conditions), to fully model and understand the complexity of AD found in clinical settings (Ranasinghe et al., 2022).

To model amyloid and P-tau pathologies we used ICV injection of Aβ and rTg4510 transgenic mice, respectively (Peña-Ortega, 2013, 2019; Torres-Flores and Peña-Ortega, 2022; Xolalpa-Cueva et al., 2022). AD-like cognitive alterations induced by ICV administration of Aβ have been previously demonstrated by several groups (Haghani et al., 2012; Peña-Ortega, 2013, 2019; Eslamizade et al., 2015), including ours (Peña-Ortega, 2013, 2019; Martínez-García et al., 2021; Torres-Flores and Peña-Ortega, 2022). On the other hand, rTg4510 transgenic mice have been useful to analyze the pathophysiology of tauopathies (Ramsden et al., 2005; SantaCruz et al., 2005; Xolalpa-Cueva et al., 2022) and the interaction between Aβ and P-tau (Busche et al., 2019). However, it is important to be cautious in ascribing the findings in rTg4510 mice only to the expression of transgenic tauP301L, since endogenous genome disruption caused by random insertion of this transgene contributes to the model’s neuropathology (Gamache et al., 2020). Moreover, we have to be aware that this mutation was identified in patients with fronto-temporal dementia with Parkinsonism linked to chromosome 17 (Yue et al., 2011) and thus is not directly linked to AD. Despite the limitations of these experimental tools, we compared both models using the hippocampal-dependent spatial memory HW maze (Rogers and Kesner, 2003; Lee and Kesner, 2004; Jerman et al., 2006; Vago et al., 2007; Hunsaker et al., 2008; Churchwell et al., 2010; Vidal-Infer et al., 2012; Boutet et al., 2018; Méndez-Salcido et al., 2022) and by analyzing hippocampal physiology (Balleza-Tapia et al., 2010; Peña et al., 2010; Alcantara-Gonzalez et al., 2019, 2021). Despite that we did not thoroughly evaluate the animals histopathologically, using Nissl staining we did not find any significant change in hippocampal CA1 integrity in the presence of Aβ, P-tau or EpoD in our experimental conditions, which contrasts with previous reports of them inducing neuronal damage (SantaCruz et al., 2005; Zussy et al., 2013; Sharma et al., 2016; Raj et al., 2019; Clark et al., 2020), in some cases associated to cognitive deficits (SantaCruz et al., 2005; Eslamizade et al., 2015; Sharma et al., 2016; Raj et al., 2019) or to changes in CA1 excitability (Eslamizade et al., 2015).

The differential contributions of either the CA3-dentate gyrus circuit or the CA1 hippocampal subregion, as well as their inputs, to each state of the HW test, have been well established (Lee and Kesner, 2004; Jerman et al., 2006). Dentate gyrus lesions impair proper encoding of information required for learning this maze on day one, as animals exhibited low EI (Lee and Kesner, 2004), which is reproduced by lesioning CA3 (Lee and Kesner, 2004; Jerman et al., 2006). In contrast, CA1 lesions reduce performance at the beginning of day two when retrieval of the information that was coded the previous day is necessary, with animals exhibiting a low RI (Vago et al., 2007; Churchwell et al., 2010). Moreover, lesions of the TA pathway specifically impair consolidation and retrieval of spatial memory in the HW maze (Vago et al., 2007). In this study, we observed that all animals learned to solve the HW maze after 3 days of repetitive training (Supplementary Figure 1A_3_), with only Tg mice exhibiting significantly poor performance during the second day (Figure 3A). However, some pathological conditions tested produced mild alterations in memory retrieval (low RI_D1-D2_), except for the combination of Aβ and P-tau pathology, which led to poor encoding on the first day of exposure to the HW maze. Thus, it is plausible that the cellular substrates of memory retrieval alterations observed in this study could mostly reside in changes in the intrinsic and synaptic properties of CA1 principal neurons and their input from the TA pathway (Vago et al., 2007). In contrast, the cellular basis for the encoding alterations in Tg mice with global Aβ, also observed in this study, could reside in deficits in the CA3 and dentate gyrus circuitry (Lee and Kesner, 2004; Jerman et al., 2006). Furthermore, we cannot exclude that the cognitive alterations observed in this study also involve pathological changes in the medial prefrontal cortex, which we have shown to be highly sensitive to Aβ-induced pathology (Torres-Flores and Peña-Ortega, 2022). Further studies will be required to demonstrate alterations in CA3 neurons and their synapses in the presence of P-tau and Aβ.

Here we found that global Aβ and P-tau pathology induce complex, differential, and even antagonistic effects on CA1 intrinsic and synaptic properties. Namely, Aβ increases the intrinsic excitability of CA1 neurons and reduces their TA synaptic inputs. In contrast, P-tau pathology induces the hypoexcitability of CA1 neurons and increases their TA synaptic inputs. The effects of Aβ in our study are similar to those found after Aβ intraparenchymal administration, which induces excitatory effects through a reduction in somatic diameter and an increase in Rm (Eslamizade et al., 2015), leading to increased firing rates (Eslamizade et al., 2015; Torres-Flores and Peña-Ortega, 2022). Moreover, Aβ disrupts microtubule integrity in axon initial segments (Zempel et al., 2017) and alters protein sorting functions and neuronal polarization (Tsushima et al., 2015), which can account for the abnormal AP waveform described in the present work. The hypoexcitability found in rTg4510 CA1 neurons, which was associated with a more depolarized firing threshold potential, has also been described in animal models of tauopathies in the hippocampus (Hatch et al., 2017) and entorhinal cortex (Booth et al., 2016a).

The depression in the TA pathway caused by global Aβ is similar to the reduction of synaptic transmission at CA3-CA1 synapses induced by either intracisternal (Alcantara-Gonzalez et al., 2019) or intraparenchymal injection of Aβ (Stéphan et al., 2001). ICV Aβ injection increases hippocampal extracellular glutamate (Raj et al., 2019), inducing a reduction in synaptic transmission (Cullen et al., 1996), possibly due to the promotion of long-term depression (LTD; Salgado-Puga et al., 2017; Torres-Flores and Peña-Ortega, 2022) and/or dendritic spine loss (Shrestha et al., 2006). In contrast, as previously shown for TA synapses (Booth et al., 2016b), P-tau pathology in rTg4510 mice enhanced synaptic transmission (Figure 6), which is similar to the increase in synaptic transmission after the injection of tau to squid giant synaptic boutons that increases intracellular calcium (Moreno et al., 2016). Indeed, the Aβ- and P-tau-induced alterations in STP can be explained by changes in release probability from TA terminals (Jackman et al., 2016). They can also be explained by shifts in GABAergic modulation, specifically the one involved in feed-forward inhibition, which modifies STP in the TA synapse (Booth et al., 2016b). Alternatively, Aβ-induced (Stéphan et al., 2001) and P-tau-induced pathology can activate glial cells (Koller and Chakrabarty, 2020), influencing synaptic plasticity (Schafer et al., 2013). Another possibility is that the mixed effects of tau pathology and global Aβ on CA1 pyramidal neurons and TA synapses are produced by homeostatic mechanisms that counterbalance the shifts in the excitability levels of the hippocampal circuit (Turrigiano and Nelson, 2004). However, the alterations in synaptic transmission caused by P-tau pathology in our Tg mice should be synapse-specific, since synaptic transmission is decreased in CA3-CA1 synapses (Booth et al., 2016b), but is enhanced in TA synapses (present work). Compensatory increases in the intrinsic excitability of CA1 pyramidal neurons can be caused by LTD induction to the CA3-CA1 synapses (Gasselin et al., 2017), whereas the opposite effect is observed if long-term potentiation is induced in this synapse (Fan et al., 2005). These changes in intrinsic excitability depend on the modulation of the postsynaptic I_h_ current, which in turn changes the sag ratio and membrane input resistance (Fan et al., 2005; Gasselin et al., 2017). TA synapses can undergo structural changes that appear to be compensatory against the presence of synapse loss at distal dendritic spines of CA1 pyramidal neurons in the 5xFAD AD model (Neuman et al., 2015). In line with this observation, the induction of LTD at TA synapses also increases the intrinsic excitability of CA1 pyramidal neurons through K^+^ currents modulation and threshold potential changes (Kim et al., 2022). Further studies will determine if the homeostatic plasticity that links the TA pathway and CA1 pyramidal neuron excitability is impaired by Aβ or P-tau pathology and if the I_h_ current is an effector of this homeostatic modulation.

As discussed, in the present work we describe opposing effects of Aβ and P-tau on intrinsic and synaptic CA1 properties. But the outcome of tau and Aβ combined was more complex than their individual effects. It appears that the excitatory effects of tau and Aβ were stronger than their inhibitory ones. These observations are consistent with reports of divergent (Tackenberg and Brandt, 2009) yet synergistic consequences of tau and Aβ on brain function (Busche et al., 2019), which appear to occur through their interactions in different neuronal compartments or organelles, like postsynaptic densities (Ittner and Götz, 2011) and mitochondria (Rhein et al., 2009). Regarding the opposite effects of tau and Aβ on intrinsic excitability, it is possible that the Aβ-induced potentiation of sodium (Ciccone et al., 2019), calcium (Haghani et al., 2012), and non-specific cationic channel function (Eslamizade et al., 2015) could override the hypoexcitability in Tg neurons, which in turn depends on the more distal position of the axon initial segment of pyramidal neurons (Hatch et al., 2017). Thus, the potentiation of these channels in the presence of Aβ could hyperpolarize the firing threshold of Tg neurons (Figure 4), which is coherent with previous reports of the excitatory mechanisms of Aβ oligomers in hippocampal slices (Tamagnini et al., 2015). We also found opposing effects of Aβ and P-tau on the sag potential (Supplementary Figure 2). The reduction of the sag potential induced by Aβ contradicts a previous report (Eslamizade et al., 2015) that used a different Aβ injection method (intracortical), which could account for the observed differences. Other studies using different experimental conditions report similar findings to ours regarding the complex antagonistic actions of Aβ on tau-induced effects (Yetman et al., 2016; Angulo et al., 2017; Busche et al., 2019; Ranasinghe et al., 2022; Barendrecht et al., 2023; Capilla-Lopez et al., 2023). Moreover, several of these reports found that the independent effects of Aβ or tau are not necessarily observed when combined (Yetman et al., 2016; Barendrecht et al., 2023; Capilla-Lopez et al., 2023), which is a reflection of the complex cross-talk between these pathological markers.

Despite the divergent or even antagonistic effects of Aβ and P-tau on CA1 excitability, both produce a similar behavioral outcome: a deficit in memory retrieval. In line with this observation, it has been shown that an overactive or hypoactive hippocampus reduces learning and memory (Zarrindast et al., 2002). A previous report described memory consolidation and retrieval impairments induced by intracortical injection of Aβ oligomers which are related to functional alterations in the hippocampus (Eslamizade et al., 2015). We have recently shown that intracortical Aβ oligomer administration induced an alteration in learning and memory that was related to hyperexcitable pyramidal neurons (Torres-Flores and Peña-Ortega, 2022). rTg4510 mice also exhibited less retention in the referenced version of the Morris water maze (Yue et al., 2011), which was associated with reduced power of hippocampal activity and reduced theta-gamma cross-frequency coupling (Booth et al., 2016b) in connection with poor spatial coding of place cells (Booth et al., 2016b).

In this work, most of the Aβ and P-tau-induced alterations in memory and hippocampal physiology were rescued by Epo-D, strongly suggesting that increasing microtubule stability could prevent several pathophysiological phenomena that drive neuronal dysfunction and dementia in AD. Specifically, our results argue that Epo-D exerts a protective role in memory consolidation and retrieval (Brunden et al., 2010; Guo et al., 2020). The reported mechanisms of Epo-D protective roles in AD models are the restoration of total microtubule bundle density (Brunden et al., 2010; Zempel and Mandelkow, 2012), improvement of mitochondrial transport along the axon (Guo et al., 2020), and normalization of axon initial segment position (Hatch et al., 2017). The cellular mechanisms behind the Epo-D-induced protective effects on membrane excitability could also include the poorly understood crosstalk between the expression and functions of ion channels and cytoskeleton dynamics (Steele and Fedida, 2014).

Importantly, our results also indicate that shifting microtubule dynamics with pharmacological tools induces complex negative and protective effects depending on the brain state. We found that Epo-D affected healthy WT mice, their cognitive flexibility, slightly altering the intrinsic excitability of their CA1 pyramidal neurons, and dramatically reducing the synaptic transmission and plasticity of their TA synapses. In contrast, WT mice subjected to global Aβ exhibited a different response, as Epo-D reduced the intrinsic excitability of their CA1 pyramidal neurons (that was augmented by Aβ) and increased their synaptic function (that was reduced by Aβ), thus normalizing hippocampal function, which was related to the rescue of memory retrieval. In the presence of P-tau pathology, Epo-D robustly increased intrinsic excitability (depressed by P-tau pathology) but worsened the alterations in STP at TA synapses. Nevertheless, Epo-D rescued the memory deterioration induced by P-tau pathology.

It is well known that neuronal microtubules play a central role in neuronal physiology (Peña-Ortega et al., 2022), including ion channel expression (Steele and Fedida, 2014), membrane excitability (Steele and Fedida, 2014), and synaptic plasticity (Waites et al., 2021). Therefore, it is expected that shifts in microtubule stability states drive complex and mixed downstream outcomes (Uchida et al., 2014; Yousefzadeh et al., 2021). For instance, shifting microtubule dynamics has clear cognitive and behavioral consequences (Uchida et al., 2014; Yousefzadeh et al., 2021; Peña-Ortega et al., 2022). It has recently been established that stable microtubules are necessary for proper consolidation and retrieval of information (Uchida et al., 2014; Yousefzadeh et al., 2021), whereas dynamic microtubules are critical for information acquisition and encoding (Uchida et al., 2014; Atarod et al., 2015). Here we found that Epo-D-treated mice exhibit impaired behavioral flexibility when learning a new maze. To the best of our knowledge, this is the first report of divergent effects of an MSA on memory and flexibility (Peña-Ortega et al., 2022). Brain processes underlying memory extinction promote cognitive flexibility, as extinction mechanisms increase performance during the learning of new tasks (Alcalá et al., 2020). Thus, it is plausible that the consolidation-enhancing effects of microtubule stabilizers could interfere with the extinction mechanisms that promote the learning of novel experiences (Uchida et al., 2014; Yousefzadeh et al., 2021), which are necessary for memory flexibility.

Hence, it can be expected that administration of MSAs can produce both beneficial effects on memory retrieval and detrimental consequences on memory flexibility in AD models and perhaps in AD patients. In this scenario, modulating the microtubule pools in a specific manner would be needed to avoid the negative outcomes on behavior. However, current pharmacological strategies lack this modulation. Pharmacological destabilization of microtubules also results in cognitive (Fanara et al., 2010) and physiological alterations (Babu et al., 2020), which have mixed effects on neural activity (Schappacher et al., 2019). Therefore, we argue that an optimal memory system requires a homeostatic range of dynamic and stable microtubule pools that efficiently respond to ongoing cognitive demands, and that such a microtubule system should be differentially affected by Aβ, P-tau and other pathological or pharmacologic modulators that drive complex or mixed physiological and behavioral consequences.

It is important to acknowledge that our study lacks an extensive microtubular and histopathological characterization of the effects of Aβ, P-tau, their combination, and Epo-D treatment, which needs to be performed. All these pathological conditions modulate synaptic density and dendritic complexity (Xolalpa-Cueva et al., 2022) and change microtubule stability (Qu et al., 2017). For instance, there is evidence that acute Aβ exposure causes microtubule stabilization and that a MSA can induce tau hyperphosphorylation and spine loss in cultured hippocampal neurons (Qu et al., 2017). Thus, these possibilities should be tested, and we intend to fill in these gaps in the near future.

## Data availability statement

The raw data supporting the conclusions of this article will be made available by the authors, without undue reservation.

## Ethics statement

The animal study was approved by Local Research Ethics Committee (INB-UNAM). The study was conducted in accordance with the local legislation and institutional requirements.

## Author contributions

ÁAR-G: conceptualization, methodology, formal analysis, investigation, writing – original draft, and visualization. BO: investigation, data curation, and writing – review and editing. J-JL-H: investigation, data curation, and writing - review and editing. FP-O: conceptualization, supervision, resources, funding acquisition, and writing – review and editing. All authors contributed to the article and approved the submitted version.
