# Supplementary material for: Deleterious and protective effects of epothilone-D alone and in the context of amyloid β- and tau-induced alterations
Source: Front Mol Neurosci. 2023 Oct 12;16:1198299. doi: 10.3389/fnmol.2023.1198299 (PMC10603193; doi:10.3389/fnmol.2023.1198299)
Supplement: Supplementary file 1 [file Presentation_1.pdf]

Supplementary Information

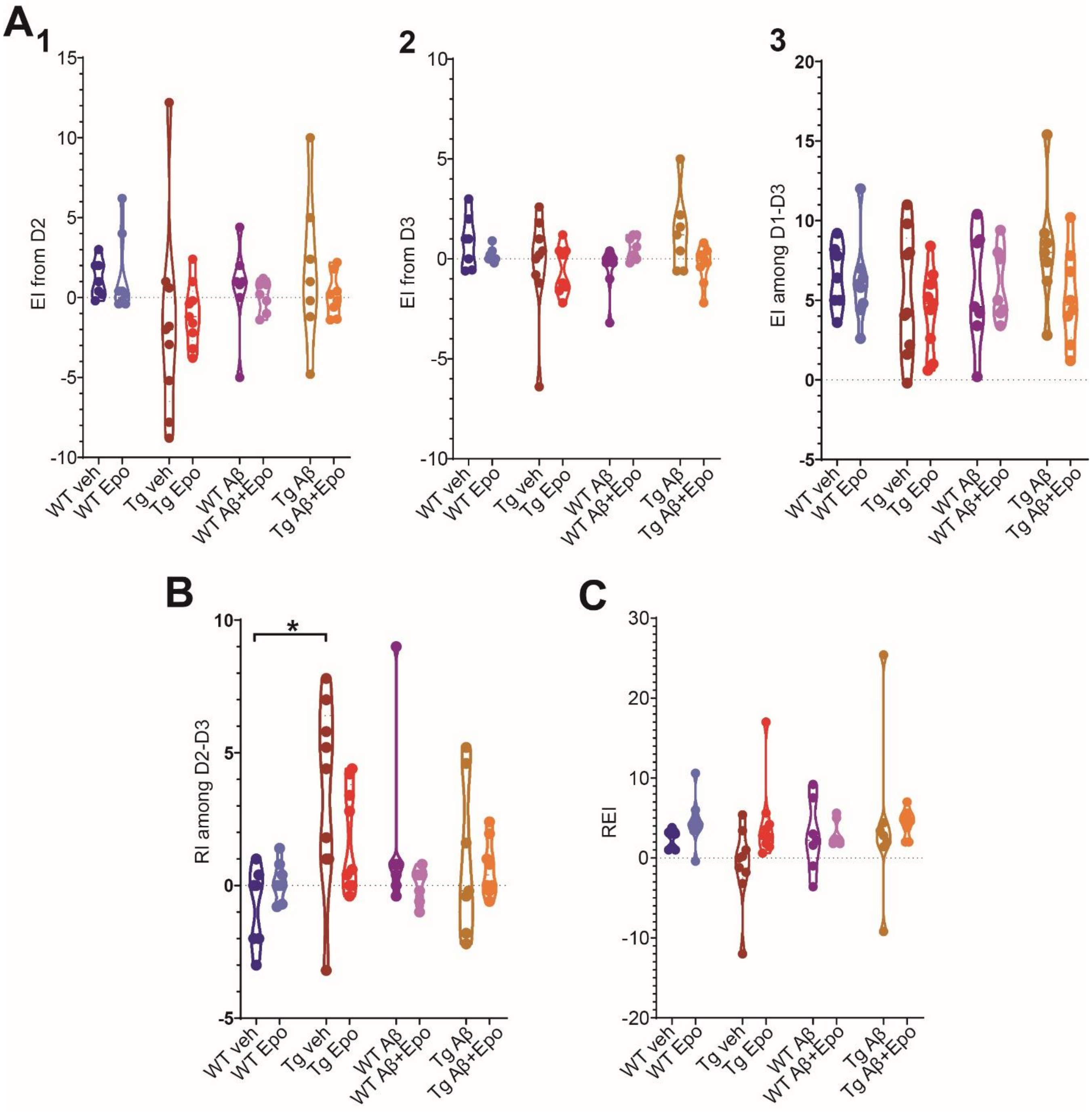

Supp. Figure 1. **A**, Quantification of the encoding indexes (EI) for days 2 (**1**) and 3 (**2**) as well as the overall encoding from day 1 to 3 (**1**). **B**, Quantification of the retrieval index (RI) on day 3 compared to day 2. **B**, Quantification of the re-encoding index (REI) on day 4 (see Material and

Methods). Abbreviations: WT = wild-type mice, Epo = Epothilone-D, Aβ = amyloid-β, Tg = transgenic mice, veh = vehicle. Asterisk denotes significant difference with a * $p < 0.05$.

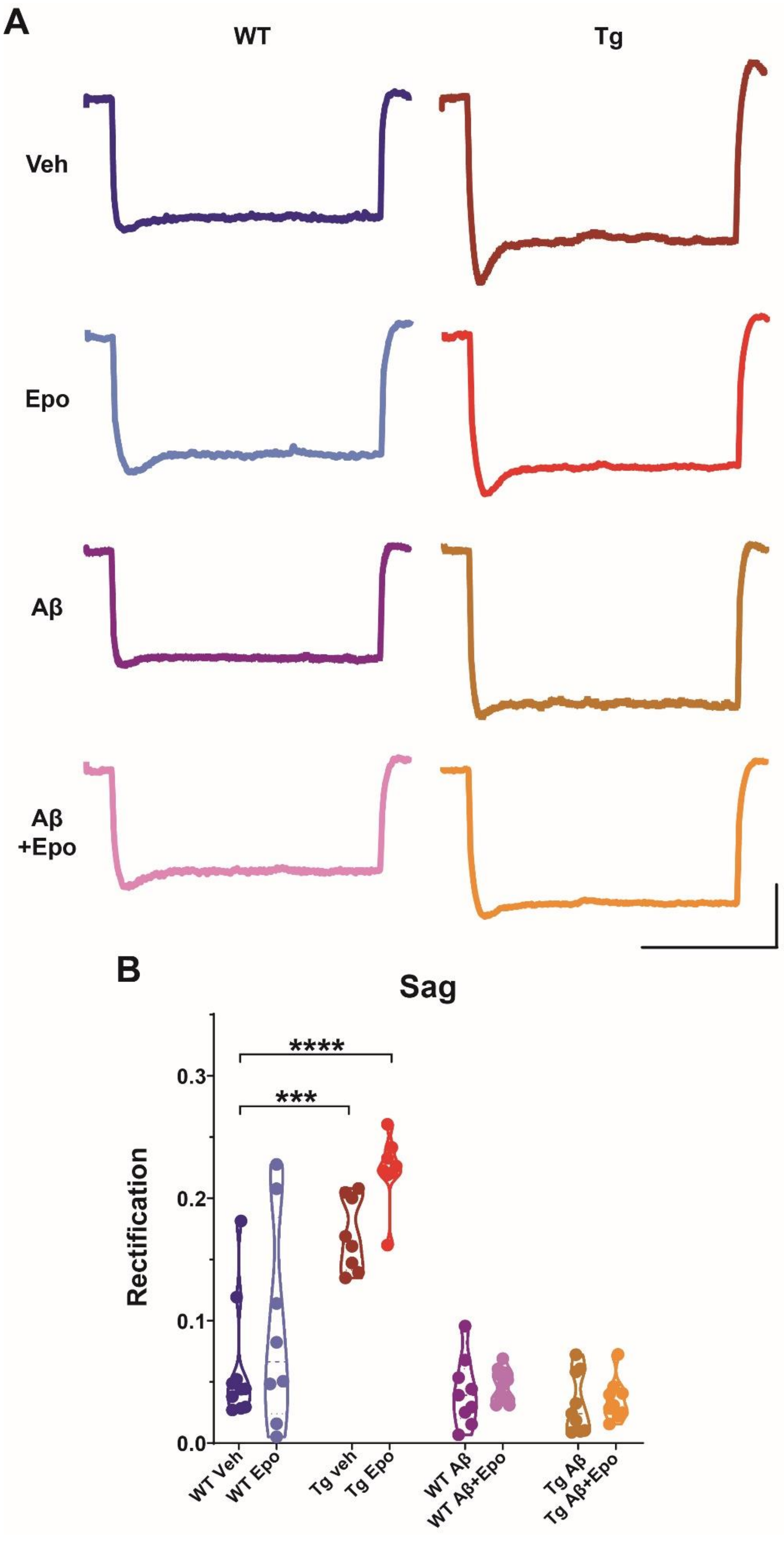

A
WT
Tg
Veh
Epo
Aβ
Aβ
+Epo
B
Sag
Rectification
0.0
0.1
0.2
0.3
****
***
WT Veh
WT Epo
Tg veh
Tg Epo
WT Aβ
WT Aβ+Epo
Tg Aβ
Tg Aβ+Epo

Supp. Figure 2. Sag potential. **A**, Representative voltage responses of CA1 principal neurons to a -200 pA square current step in all experimental groups. Scales: 500 ms, 15 mV. **B**, Quantification of the rectification proportion obtained as the quotient of the initial peak voltage response divided by the voltage at the end of the response (i.e., sag potential; see Materials and Methods). Abbreviations: WT = wild-type mice, Epo = Epothilone-D, Aβ = amyloid-β, Tg = transgenic mice, veh = vehicle. Asterisks denote significant differences as follows: *** $p < 0.001$, **** $p < 0.0001$.

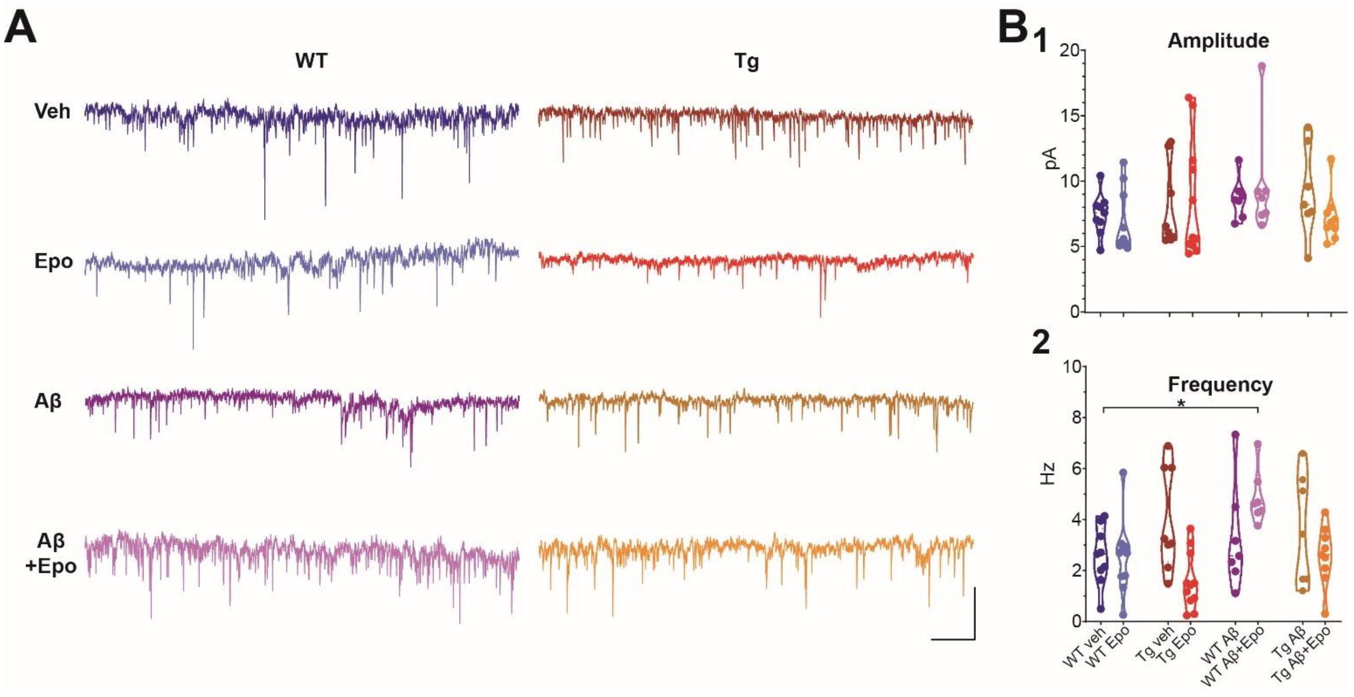

Supp. Figure 3. Global spontaneous synaptic activity. **A**, Representative traces of spontaneous postsynaptic currents recorded in CA1 principal neurons from all experimental groups. Scales: 2 s, 10 pA. **B**, Quantification of mean (**1**) amplitude and (**2**) instantaneous frequency of spontaneous postsynaptic currents. Abbreviations: WT = wild-type mice, Epo = Epothilone-D, Aβ = amyloid-β, Tg = transgenic mice, veh = vehicle. Asterisk denotes significant difference with a * $p < 0.05$.
